# Global, regional, and national burdens of cancer in children aged zero to nine years from 1990 to 2019

**DOI:** 10.7189/jogh.14.04104

**Published:** 2024-05-31

**Authors:** Ping Wang, Shu Huang, Xiaomin Shi, Huan Xu, Ruiyu Wang, Jieyu Peng, Qi Chen, Wei Zhang, Lei Shi, Xian Zhou, Xiaowei Tang

**Affiliations:** 1 Department of Gastroenterology, Affiliated Hospital of Southwest Medical University, Luzhou, China; 2 Nuclear Medicine and Molecular Imaging Key Laboratory of Sichuan Province, Luzhou, China; 3 Department of Gastroenterology, Lianshui County People’ Hospital, Huaian, China; 4 Department of Gastroenterology, Lianshui People’ Hospital of Kangda College Affiliated to Nanjing Medical University, Huaian, China

## Abstract

**Background:**

The description of long-term trends in the cancer burden among children aged zero to nine years from 1990 to 2019 reveals significant changes in children’s health. It helps in resource allocation and health policy planning. We analysed data on the incidence, mortality, and disability-adjusted life years (DALYs) by sex and age group in children aged zero to nine.

**Methods:**

Estimates of DALYs for children aged zero to nine years, appeared as part of the Global Burden of Diseases, Injuries, and Risk Factor Study 2019, by age, sex, and location for 1990–2019. We also provided estimations by the sociodemographic index (SDI) quintile, a systematic measure to indicate educational attainment, income per capita, and total fertility rate for those younger than 25 years. We used age-period-cohort models to investigate paediatric cancers prevalence, incidence, mortality, and DALYs rates and auto-regressive integrated moving average models to predict cancer in children of different age groups in males and females.

**Results:**

A total of 6 224 010 DALY numbers for cancer cases occurred globally in 2019 among children aged zero to nine years. Additionally, the incidence of paediatric cancers in 2019 in the middle SDI countries was the highest, including 60 662 cases, and the highest mortality and DALYs cases of paediatric cancers were in the low SDI countries (25 502 and 2 199 790). The joinpoint regression analysis revealed that the trend of total cancer burden in age-standardised mortality rates and age-standardised DALYs rates showed a significant decrease with an average annual percentage change of –2.10 and –2.03 from 1990 to 2019. Furthermore, the paediatric cancer spectrum was changing. Other malignant neoplasms and other leukaemia were the major components of cancer in all age groups of children.

**Conclusions:**

The disease burden in children aged zero to nine years decreased significantly globally from 1990 to 2019. However, the overall prediction of childhood cancer increased slightly from 2020 to 2040. Our findings may help guide investments and inform policies. This highlights the necessity to improve current treatment measures and establish effective prevention strategies to reduce the cancer burden among children aged zero to nine years.

Cancer is the second leading cause of mortality worldwide and is currently a major threat to global development [1]. Children with cancer often die without a timely and accurate diagnosis and treatment, and there are no evidence-based and risk-reduction tactics available to improve outcomes [2]. A recent study based on the Global Burden of Diseases, Injuries, and Risk Factor Study 2019 (GBD 2019) showed that the global burden of children’s cancer had remained markedly high in four indicators – incidence, prevalence, deaths and disability-adjusted life years (DALYs) – from 1990 to 2019, particularly in the low-middle sociodemographic index (SDI) regions [3]. Children with cancer receive better diagnosis and treatment in developed countries. Therefore, the percentage of children surviving for five years in developed countries is higher than in developing countries that lack medical resources [4]. In developed countries, the change in the cancer spectrum helps to establish a cancer control system and contributes to planning and enacting strategies [5]. However, few resources are used to prevent cancer and change the trend of cancer in developing countries [6,7]. Several factors, such as ecological, environmental, demographic, cultural, and genetic factors, result in changes in cancer incidence, mortality, and DALYs burden [8,9].

The majority of cancer DALYs among children younger than 19 years affect countries with a lower SDI [9]. Adolescent and young adult cancer in people aged 15–39 years contributed to 23.5 million (95% uncertainty interval (UI) = 21.9, 25.2) DALYs to the global burden of disease in 2019, of which 2.7% (95% UI = 1.9, 3.6) came from years lived with disabilities (YLDs) and 97.3% (95% UI = 96.4, 98.1) from year of life lost (YLLs) [10]. Further, the high SDI regions have the highest age-standardised incidence rate (ASIR) but the lowest age-standardised DALYs rate [10]. The DALYs burden of adult cancer is heavy in countries with high and middle SDI settings, which is probably due to the older population and higher lifestyle risk factors [9,10]. The difference in the epidemiological patterns of cancer burden between children and adults demonstrates that the current policy principally concentrates on risk reduction and intervention measures for adults [9]. Thus, early diagnosis and effective treatments are required from efficient health systems to improve the prognosis and reduce the burden of cancers in children [11].

Children are often neglected when administrations formulate cancer control policies in low- and middle-income countries [12]. A recent study illustrated that leukaemia and brain and nervous system cancer contributed to the greatest proportion of DALYs burden in all child age groups (zero to four, five to nine, and 10–14 years). Further, the age group of zero to four years had the greatest contribution to global children’s cancer DALYs (n = 4.3 million; 95% UI = 3.8, 4.7), or 37% (95% UI = 36.9, 37.0) of the global children’s cancer absolute DALYs burden (in the age group of zero to 19 years) according to GBD 2017 [13]. However, these studies focused on the global burden of childhood cancer in the broad age group of zero to five, zero to 19, and 15–39 years, not including the age group under nine years. The leading cancer types of children cancer varied across age groups, and some cancers were more prevalent in specific age groups. Thus, heterogeneity in the cancer burden among children aged zero to nine years should not be ignored. Updated and accurate data from the GBD 2019 are crucial for the administration to estimate resource needs such as infrastructure, medicines, and health technologies, understand how many children will develop and survive cancer before delivering health services, and know what types of cancer are common among children [14].

The GBD 2019 provided a systematic estimate of 359 diseases and injuries, including cancer, which is beneficial for filling the gap on cancer in children younger than nine years [15]. To assess the global burden of cancer in children, we present the results of the GBD 2019 study for 14 cancer groups, including the cancer incidence, mortality, YLLs, YLDs, and DALYs for 204 countries and territories from 1990 to 2019. We estimated the burden (incidence, mortality, DALYs, YLLs, and YLDs) of total and specific children’s cancer globally and in different regions and nations in 2019. Moreover, we predicted the total and specific cancer burden among children younger than nine years from 1990 to 2024.

## METHODS

### Data sources

GBD was a comprehensive and comparable data source that provided metrics that included incidence, prevalence, mortality, DALYs, YLLs, and YLDs to describe different aspects of children’s cancer burden. Estimates are generated for total cancer and each cancer and are reported by age groups, sex, regions and years. General methods and original data for GBD 2019 have been described previously [16–18]. The approximate approach and structure for cancer estimation in GBD 2019 are similar to those in GBD 2017 [19]. The incidence, mortality, and DALYs attributed to cancer in 204 countries and territories from 1990 to 2019 were acquired from the GBD 2019 online results tool, run by the Institute for Health Metrics and Evaluations [20].

Data for the GBD 2019 were derived from literature reviews and identified through research collaborations, including vital registration systems, verbal autopsy data, scientific reports, and hospital and administrative population surveys. The vital registration system preserves data on crucial events in a person’s life (birth, death, and cause of mortality). For certain locations lacking a vital registration system, a verbal autopsy is an effective data collocation method in which an interviewer uses a questionnaire to acquire disease information such as signs, symptoms, and lifestyle characteristics of a recently dead person from someone acquainted with the deceased. Cancer registries generally record and administer data regarding cancer patients [16].

### Definition

Although the definition of the age range for children varies, particularly in the upper limit [16–19], we used the age range of zero to nine years since there were previous studies provided. Data for this range are accessed with the GBD Result Tool, and subsets of this age range are accessed with the GBD Compare data visualisation tool or GBD Result Tool. All malignant cancer types identified in the 10th revision of the International Classification of Diseases (chapter two – neoplasms) were included in the analysis [19]. We defined any cancer with less than 1000 global deaths annually as rare. We reorganised the cancer spectrum to demonstrate the most relevant information regarding paediatric cancers, including leukaemia (C95.2), acute lymphoid leukaemia (ALL; C91.00), acute lymphoid leukaemia (AML; C92.0), Hodgkin lymphoma (HL; C81.9), non-Hodgkin lymphoma (NHL; C85.9), other leukaemia (C95.7), brain and central nervous system cancer (CNS; D46.901), kidney cancer (C64.x00), lip and oral cavity cancer (C00.503), liver cancer (C22.0), malignant skin melanoma (C43.091), nasopharynx cancer (C11.9), ovarian cancer (C56.x00), and thyroid cancer (C73.x00) and other malignant neoplasms. Other malignant neoplasms comprise all malignancies without a separate GBD cause category listed. This category does not include non-melanoma skin cancers and myelodysplastic or myeloproliferative neoplasms, which are separate GBD-cause categories not included in this analysis. Other leukaemia included leukaemia not otherwise specified. Data were collected on these cancer types from both sexes in four age groups (<28 days, 28–365 days, one to four years, and five to nine years) and by the 21 regional groupings of countries. In this study, we used subgroups to define children aged zero to nine years, including infants (<1 year, <28 days and 28–365 days old), children aged zero to four years, and children aged five to nine years, to demonstrate their growth and comprehensive understanding of this life phrase.

### Mortality and no-fatal estimations of cancer burden

From the GBD 2019, we obtained repeated cross-sectional data from the Global Health Data Exchange, which contains the global burden of 369 diseases and injuries, including cancer, in 204 countries and territories from 1990 to 2019. DisMod-MR 2.1, a Bayesian mixed-effects meta-regression tool, generated an interior estimation for the prevalence, incidence, and cause-specific mortality at five-year intervals, subsequently interpolated to generate annual estimations. We used a standard cause of death ensemble model (CODEm) method to evaluate mortality caused by cancers. The GBD 2019 study starts with mortality estimations from multiple programmes. Data from cancer registries may be scarce, although incident data are more readily available. Thus, to maximise data availability in locations with unreliable mortality information, mortality-to-incidence (MIRs) were used to convert cancer registry incidence data to mortality estimations. The final MIRs for age, sex, location, and year were processed by spatiotemporal Gaussian process regression and multiplied with the incidence data to generate crude mortality data [21]. Because mortality data are unavailable for every age, sex, location, and year combination, CODEm is necessary to estimate mortality data.

The estimates of the YLLs were calculated using reference life tables and the estimated mortality generated from CODEm [1]. The 10-year prevalence was divided into four fixed durations: diagnosis and primary phase, metastatic phase, terminal phase, and the remaining duration assigned to the controlled phase [19]. Specific YLDs were calculated by multiplying the specific prevalence with disability weights. The DALY estimates were the sum of YLDs and YLLs. One DALY meant one year of healthy life was lost. All rates are shown per 100 000 individuals The final estimate was indicated by 95% UIs. The UIs were calculated at each step in the cancer estimation process by drawing the 2.5th and 97.5th percentiles of the distribution of 1000 draws based on the GBD algorithm [21].

### Statistical analysis

The first aim of this study was to show the current and long-term trends in total cancer incidence, mortality, and DALYs in children aged zero to nine years according to sex. The recognition of changes in time trends is a crucial problem in the analysis of incidence, mortality, and DALY data of paediatric cancers, and such research has been indicated by a joinpoint regression model [22]. The annual percentage change (APC) model is based on the Poisson distribution and can demonstrate general trends in paediatric cancers by age, period, and cohort. To determine the magnitude of the time trend for prevalence, incidence, mortality, DALY rate, the average annual percent change (AAPC), and the corresponding 95% confidence interval (CI) were calculated using joinpoint regression analysis. The AAPC is an overall indicator of the trend by a preset fixed interval. It is calculated as a weighted average of the APC, a single number describing the means of APCs over the past decades. The APC was computed using the geometrically weighted average of the various annual percentage changes in the regression models. A positive APC value reveals an increasing trend in prevalence, incidence, mortality, and DALYs rate; conversely, a negative value indicates a decreasing trend. The model used the age-specific cancer rates as the dependent variable and the year as the dependent variable to produce the corresponding log-line model, showing the breakpoint of joinpoint to indicate the trend of disease change over time and determine whether the change trend in each segment was statistically significant. This model was performed by joinpoint, version 4.9.1.0 (National Cancer Institute, Rockville, Maryland, USA). The Nordpred model is a long-term prediction model based on the age-period-cohort model [23]. We predicted the long-term trends in the incidence rate of cancer in children of different age groups (zero to four and five to nine years) by sex from 1990 to 2040.

## RESULTS

### Global paediatric cancer burden

**Figure 1:**
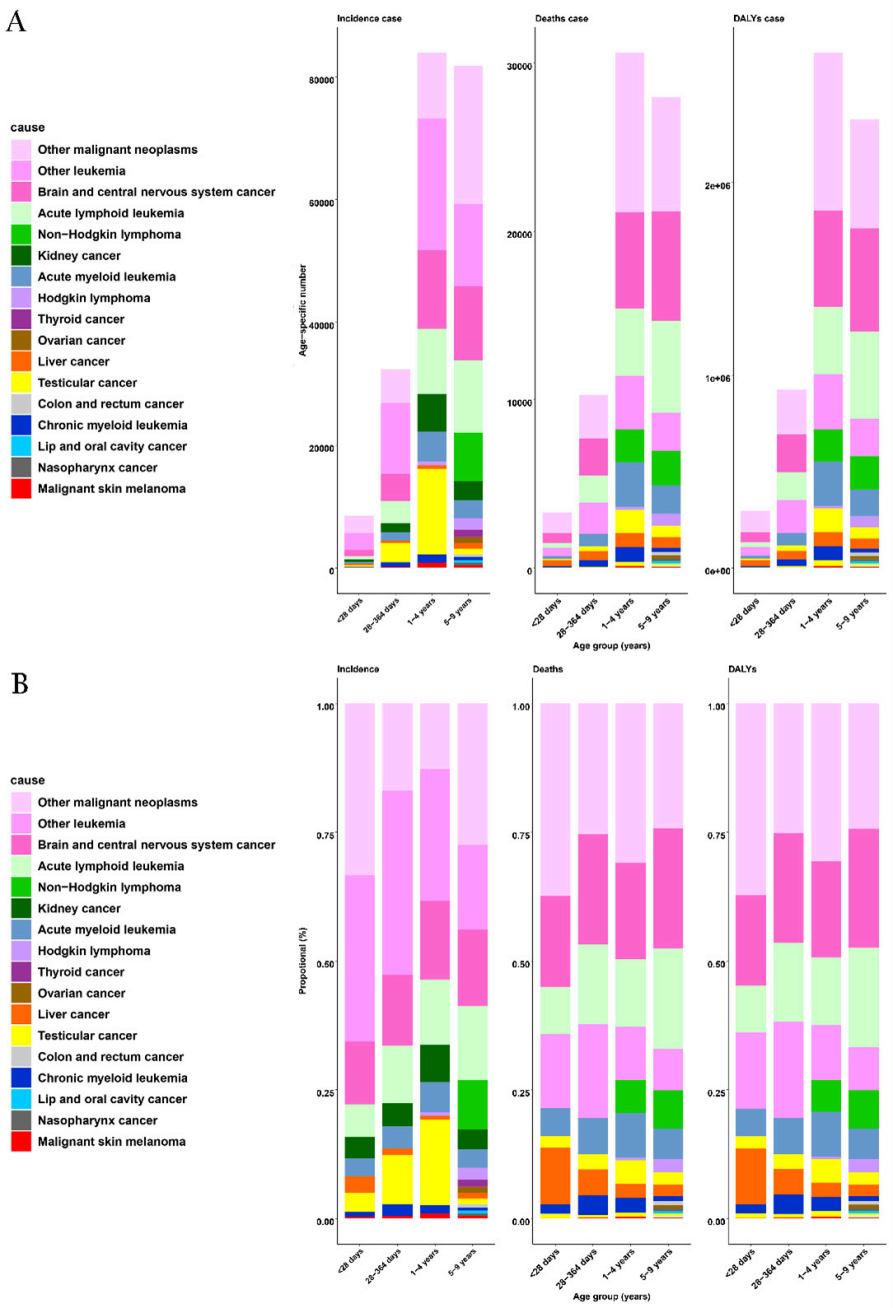
Global burdens of children cancer types by age group, in 2019, for both sexes combined. **Panel A.** Global age-specific incidence, deaths and DALY numbers. **Panel B.** Proportional incidence, deaths, DALYs. Other malignant neoplasms comprise all malignancies without a separate GBD cause category listed. This category does not include non-melanoma skin cancers and myelodysplastic or myeloproliferative neoplasms, which are separate GBD-cause categories not included in this analysis. Other leukaemia include leukaemia not otherwise specified. DALYs – disability-adjusted life years, GBD – Global Burden of Disease, Injuries, and Risk Factor Study

The main causes of mortality among children aged zero to nine years were chronic respiratory diseases, respiratory infections, tuberculosis, and enteric infections. Cancer had low values and a stable trend (Figure S1 in the **Online Supplementary Document**). However, an estimated incidence of cancer cases (n = 206 602; 95% UI = 175 253, 240 576), mortality cancer cases (n = 72 190 (95% UI = 61 735, 84 353), DALYs cancer cases (n = 6.2 million; 95% UI = 5.3, 7.3) occurred globally in 2019 among children aged zero to nine years (**Table 1**). In terms of sex, the incidence, mortality, and DALYs of cancer in children were higher among boys than girls (Tables S1–2 in the **Online Supplementary Document**). Furthermore, among all age groups, children aged one to four years had the highest cancer incidence, mortality, and DALYs, whereas children under 28 days had the lowest values of all these estimates (**Figure 1**, panel A).

**Table 1:** Worldwide incidence, deaths, DALYs, and rates by SDI and regions for total cancer in 2019 in children younger than 10 y and the change in trends from 1990 to 2019 in both sexes

	Incidence			Deaths			DALYs		
**Location**	**N (95% UI)**	**Rate per 10 000 population**	**Change in rates, % (95% UI)**	**N (95% UI)**	**Rate per 10 000 population**	**Change in rates, % (95% UI)**	**N (95% UI)**	**Rate per 10 000 population**	**Change in rates, % (95% UI)**
Global	206 602 (175 253, 240 576)	15.68 (13.30, 18.26)	–0.35 (–0.50, –0.13)	72 190 (61 735, 84 353)	5.48 (4.60, 6.40)	–0.46 (–0.58, –0.27)	6 224 010 (5 317 408, 7 300 278)	224.27 (191.60, 263.05)	–0.45 (–0.57, –0.25)
SDI									
*High*	21 919 (15 223, 30 266)	20.48 (14.23, 28.28)	–0.08 (–0.25, 0.13)	2828 (2479, 3058)	2.64 (2.32, 2.86)	–0.43 (–0.52, –0.37)	250 260 (217 820, 274 039)	112.66 (98.05, 123.36)	–0.41(–0.51, –0.35)
*High-middle*	37 056 (30 346, 44 392)	22.53 (18.45, 26.99)	–0.24 (–0.40, –0.03)	7286 (6319, 8222)	4.43 (3.84, 5.00)	–0.56(–0.64, –0.45)	634 713 (549 469, 718 642)	183.71 (159.04, 208.00)	–0.55(–0.63, –0.44)
*Middle*	60 662 (52 596, 69 056)	16.42 (14.24, 18.70)	–0.41(–0.55, –0.19)	17 541 (15 264, 19 890)	4.75 (4.13, 5.38)	–0.55 (–0.65, –0.41)	1 510 063 (1 314 513, 1 715 597)	195.29 (170.00, 221.87)	–0.54 (–0.64, –0.40)
*Low-middle*	41 812 (33 992, 50 952)	12.06 (9.80, 14.69)	–0.36 (–0.55, –0.02)	18 973 (15 559, 22 728)	5.47 (4.49, 6.55)	–0.44 (–0.59, –0.14)	1 624 084 (1 329 980, 1 945 944)	223.75 (183.23, 268.10)	–0.42 (–0.58, –0.11)
*Low*	45 006 (33 816, 57 540)	13.68 (10.28, 17.49)	–0.34 (–0.56, 0.24)	25 502 (19 462, 32 622)	7.75 (5.91, 9.91)	–0.42 (–0.60, –0.04)	2 199 790 (1 671 898, 2 812 680)	311.27 (236.57, 397.99)	–0.41 (–0.59, –0.01)
Africa									
*Central sub-Saharan*	3433 (2216, 5126)	8.56 (5.53, 12.78)	–0.52 (–0.70, 0.11)	1791 (1199, 2566)	4.47 (2.99, 6.40)	–0.55 (–0.70, –0.11)	153 746 (102 589, 220 931)	179.25 (119.61, 257.58)	–0.53 (–0.69, 0.06)
*Eastern sub-Saharan*	20 547 (13 646, 28 308)	16.71 (11.10, 23.02)	–0.42 (–0.67, 0.27)	12 230 (9184, 15 930)	9.95 (7.47, 12.96)	–0.52 (–0.70, –0.14)	1 061 332 (794 617, 1 382 698)	400.95 (300.19, 522.35)	–0.50 (–0.69, –0.11)
*North and the Middle East*	19 288 (15 797, 23 309)	16.18 (13.25, 19.55)	–0.28 (–0.50, 0.16)	5780 (4598, 6983)	4.85 (3.86, 5.86)	–0.45 (–0.61, –0.11)	496 828 (394 771, 600 391)	198.26 (157.53, 239.59)	–0.43 (–0.60, –0.08)
*Southern sub-Saharan*	1058 (844, 1330)	6.57 (5.24, 8.26)	–0.13 (–0.36, 0.19)	420 (335, 522)	2.61 (2.08, 3.25)	–0.23 (–0.43, 0.05)	35 808 (28 579, 44 568)	105.59 (84.27, 131.42)	–0.23 (–0.43, 0.07)
*Western sub-Saharan*	14 584 (9794, 20 104)	10.5 (7.05, 14.47)	–0.08 (–0.38, 0.31)	8687 (5805, 12 112)	6.25 (4.18, 8.72)	–0.22 (–0.44, 0.09)	747 613 (499 277, 1 043 628)	249.27 (166.47, 347.96)	–0.19 (–0.42, 0.12)
America									
*Andean Latin*	2134 (1532, 2829)	17.22 (12.37, 22.83)	–0.23 (–0.58, 0.22)	750 (547, 986)	6.05 (4.41, 7.96)	–0.39 (–0.65, –0.10)	64 001 (46 677, 84 482)	242.89 (177.14, 320.62)	–0.39 (–0.64, –0.09)
*Central Latin*	7212 (5066, 10 340)	16.64 (11.69, 23.85)	–0.16 (–0.40, 0.17)	2348 (1888, 2920)	5.41 (4.35, 6.74)	–0.35 (–0.49, –0.16)	201 225 (161 273, 250 716)	221.12 (177.21, 275.5)	–0.33 (–0.48, –0.14)
*High-income North*	8116 (5356, 11 786)	18.97 (12.52, 27.55)	–0.15 (–0.31, 0.07)	1200 (1089, 1284)	2.81 (2.55, 3.00)	–0.33 (–0.41, –0.27)	105 404 (94 637, 113 945)	118.57 (106.46, 128.18)	–0.31 (–0.38, –0.24)
*Southern Latin*	1899 (998, 3833)	19.04 (10.01, 38.43)	0.03 (–0.36, 0.87)	420 (374, 479)	4.21 (3.75, 4.81)	–0.41 (–0.49, –0.32)	36 222 (31 890, 41 667)	175.55 (154.55, 201.94)	–0.39 (–0.47, –0.29)
*Tropical Latin*	5319 (3971, 6788)	16.25 (12.13, 20.73)	–0.23 (–0.44, 0.01)	1950 (1553, 2359)	5.96 (4.74, 7.21)	–0.38 (–0.55, –0.19)	166 855 (132 730, 201 987)	244.71 (194.66, 296.23)	–0.38 (–0.55, –0.20)
*Caribbean*	1583 (1025, 2264)	20.11 (13.02, 28.76)	–0.21 (–0.39, 0.03)	709 (445, 1043)	9.00 (5.65, 13.24)	–0.23 (–0.41, –0.01)	60 653 (37 997, 89 077)	366.08 (229.34, 537.63)	–0.21 (–0.39, 0.02)
Asia									
*Central*	2874 (2214, 3723)	15.21 (11.72, 19.71)	–0.4 (–0.53, –0.23)	1035 (873, 1239)	5.48 (4.62, 6.56)	–0.38 (–0.50, –0.24)	88 206 (74 205, 106 240)	221.01 (185.93, 266.19)	–0.37 (–0.49, –0.22)
*East*	45 586 (37 894, 54 804)	28.6 (23.78, 34.38)	–0.38 (–0.56, –0.01)	8443 (6968, 10 137)	5.3 (4.37, 6.36)	–0.66 (–0.75, –0.50)	740 703 (609 219, 895 800)	215.79 (177.48, 260.98)	–0.65 (–0.74, –0.49)
*High-income Pacific*	3072 (2420, 3886)	20.16 (15.88, 25.5)	–0.11 (–0.29, 0.12)	377 (333, 410)	2.47 (2.19, 2.69)	–0.51 (–0.62, –0.45)	33 534 (29 376, 36 612)	107.50 (94.17, 117.36)	–0.51 (–0.62, –0.44)
*South*	34 976 (27 539, 43 728)	10.38 (8.17, 12.97)	–0.24 (–0.49, 0.22)	16 999 (13 686, 20 589)	5.04 (4.06, 6.11)	–0.38 (–0.58, 0.01)	1 450 376 (1 166 285, 1 757 960)	207.68 (167.00, 251.73)	–0.36 (–0.56, 0.05)
*Southeast*	16 078 (13 051, 20 287)	14.45 (11.73, 18.24)	–0.35 (–0.56, 0.10)	6068 (4962, 7614)	5.45 (4.46, 6.84)	–0.41 (–0.58, –0.06)	519 774 (425 418, 652 445)	225.04 (184.19, 282.48)	–0.40 (–0.58, –0.04)
Europe									
*Central*	2071 (1270, 3349)	17.90 (10.98, 28.95)	–0.15 (–0.42, 0.30)	394 (340, 460)	3.40 (2.94, 3.98)	–0.55(–0.64, –0.46)	34 232 (29 297, 40 104)	142.89 (122.29-167.4)	–0.55 (–0.64, –0.46)
*Eastern*	4312 (3121, 5849)	16.98 (12.29, 23.03)	–0.31 (–0.46, –0.12)	1104 (964, 1258)	4.35 (3.80, 4.95)	–0.53 (–0.59, –0.46)	95 247 (83 005, 108 936)	181.94 (158.56, 208.09)	–0.52 (–0.59, –0.45)
*Western*	11 184 (7284, 15 884)	24.74 (16.11, 35.14)	–0.07 (–0.27, 0.21)	1165 (991, 1280)	2.58 (2.19, 2.83)	–0.49 (–0.58, –0.43)	104 330 (87 456, 117 103)	111.6 (93.55, 125.26)	–0.47 (–0.57, –0.40)
Oceania									
*Oceania*	557 (341, 844)	16.24 (9.95, 24.6)	0.03 (–0.26, 0.46)	222 (148, 319)	6.48 (4.30, 9.31)	–0.02 (–0.30, 0.36)	19 190 (12 661, 27 709)	254.90 (168.18, 368.05)	–0.03 (–0.30, 0.35)
*Australasia*	719 (376, 1283)	19.66 (10.29, 35.07)	0.10 (–0.28, 0.70)	99 (84, 111)	2.70 (2.30, 3.04)	–0.41 (–0.51, –0.32)	8729 (7324, 9994)	113.96 (95.62, 130.47)	–0.39 (–0.51, –0.29)

DALYs – disability-adjusted life years, SDI – sociodemographic index, UI – uncertainty interval

Table S3 in the **Online Supplementary Document** shows the AAPCs of epidemiological metrics of total cancer among all ages and children aged zero to nine years. The age-standardised prevalence rate (ASPR) rose by 0.64 (95% CI = 0.57, 0.71) but declined by 0.59 (95% CI = –0.65, –0.51) for mortality for all ages over the last few decades. However, for children aged zero to nine years, the AAPC of total cancer for ASIR and ASPR decreased. The age-standardised mortality rate (ASMR) declined by 2.1% (95% CI = –2.22, –1.98). In addition, the younger group showed a more substantial decrease in ASIR and ASMR in women compared with all age groups.

**Figure 2:**
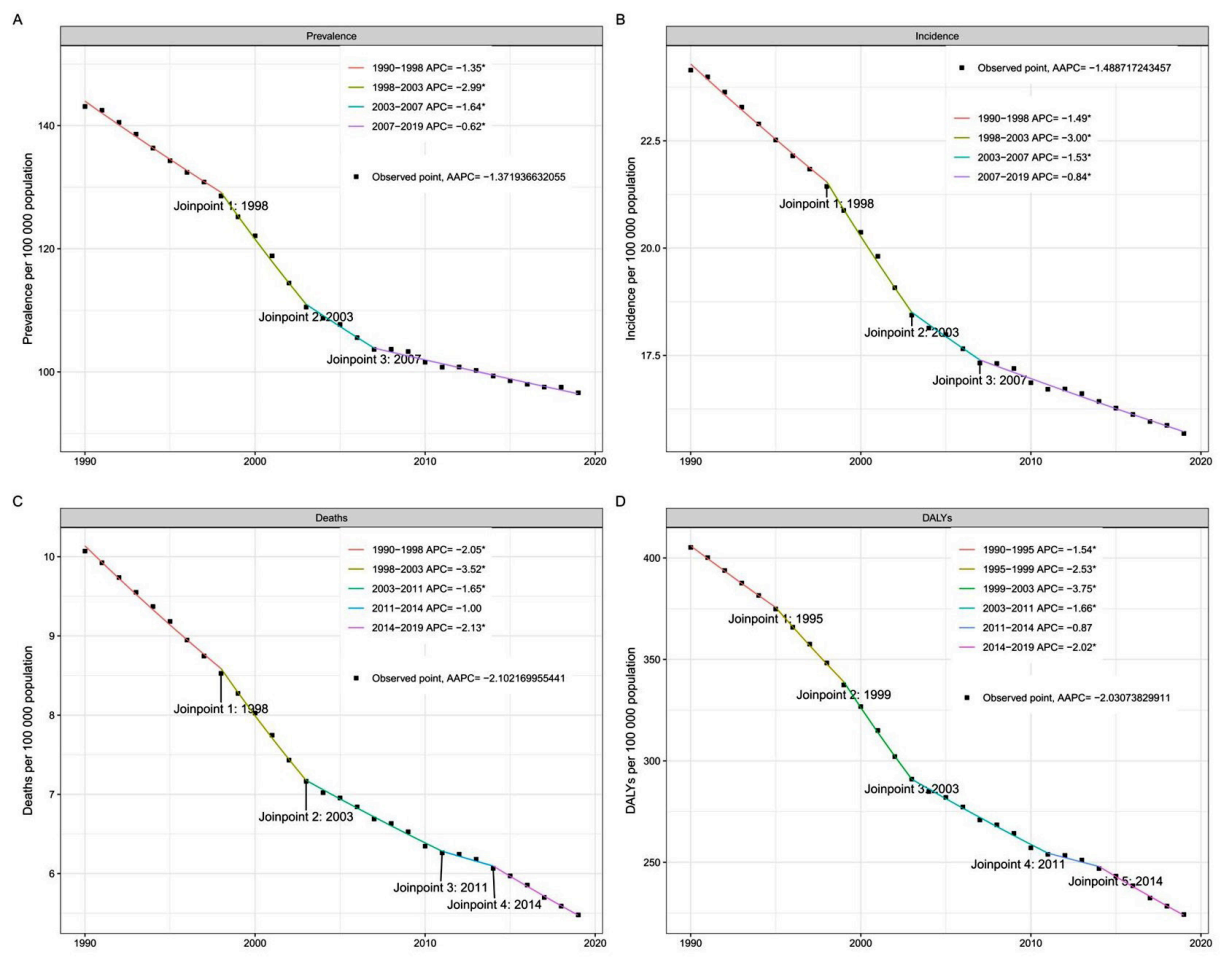
Joinpoint regression analysis of global total cancer in adolescents and young adults aged zero to nine years from 1990 to 2019. **Panel A.** Prevalence rates. **Panel B.** Incidence rates. **Panel C.** Mortality rates. **Panel D.** DALYs rates. APC – annual percentage change, DALYs – disability-adjusted life years

Over the past 30 years, the global ASPR, ASIR, ASMR, and DALY rates of children aged zero to nine years have decreased significantly. ASPR and ASIR have markedly decreased since 1990, with the most noticeable decrease observed between 1998–2003 (ASPR = –2.99, *P* < 0.05; ASIR = 3.00, *P* < 0.05) (**Figure 2**, panels A–B). However, the prevalence and incidence decreasing trends gradually stabilised between 2007–19 (ASPR = –0.62, *P* < 0.05; ASIR = –0.84, *P* < 0.05). Similarly, the ASMR and age-standardised DALY rates of paediatric cancers also significantly decreased with different APCs, which was most evident between 1998–2003 for the ASMR and 1999–2003 for age-standardised DALYs rates (ASMR = 3.52, *P* < 0.05; age-standardised DALYs rate = 3.75, *P* < 0.05) (**Figure 2**, panels C–D).

Figure S2 in the **Online Supplementary Document** predicts common cancer incidence by sex in 2040 among children aged zero to four and five to nine years. The results showed that the predicted trend of total cancer in females in both age groups was significantly higher than in males. The global trend of testicular cancer in male children aged zero to four years has gradually increased, but the trend may decrease in the future. The trend declined significantly over the past 30 years for other leukaemia, but it could slightly increase among children aged zero to four years in both males and females. Finally, for most cancers among children aged zero to four and five to nine years, the prospective trend was stable and changed little.

### Regional and national childhood cancer burden

The highest incidence and rates of paediatric cancers among children aged zero to nine years in both males and females were in East Asia (n = 45 586, 95% UI = 37 894, 54 804; rate = 28.6, 95% UI = 23.78, 34.38), followed by South Asia (n = 34 976, 95% UI = 27 539, 43 728; rate = 10.38, 95% UI = 8.17, 12.97) (**Table 1**). The highest incidence rates of total cancer in male children were in Western Europe and females in East Asia. For adults of all ages, the highest incidence rates in both males and females were in high-income North America, followed by Australasia. Nevertheless, South Asia showed the highest mortality (n = 16 999) and DALY (n = 1 450 376) cases among children with cancer in both males and females in 2019. Eastern sub-Saharan Africa showed the highest mortality and DALY rates in males and females in 2019, of which the rates for males were significantly higher than for females (Figure S3 in the **Online Supplementary Document**). Since 1990, childhood cancer incidence rates have decreased in most regions, with the greatest decrease observed in Central sub-Saharan Africa and the greatest increase observed in Oceania. As for mortality and DALY rates, all regions revealed a declining trend over the past 30 years, with the most significant decrease in East Asia (**Table 1**, Figure S3 in the **Online Supplementary Document**).

**Figure 3:**
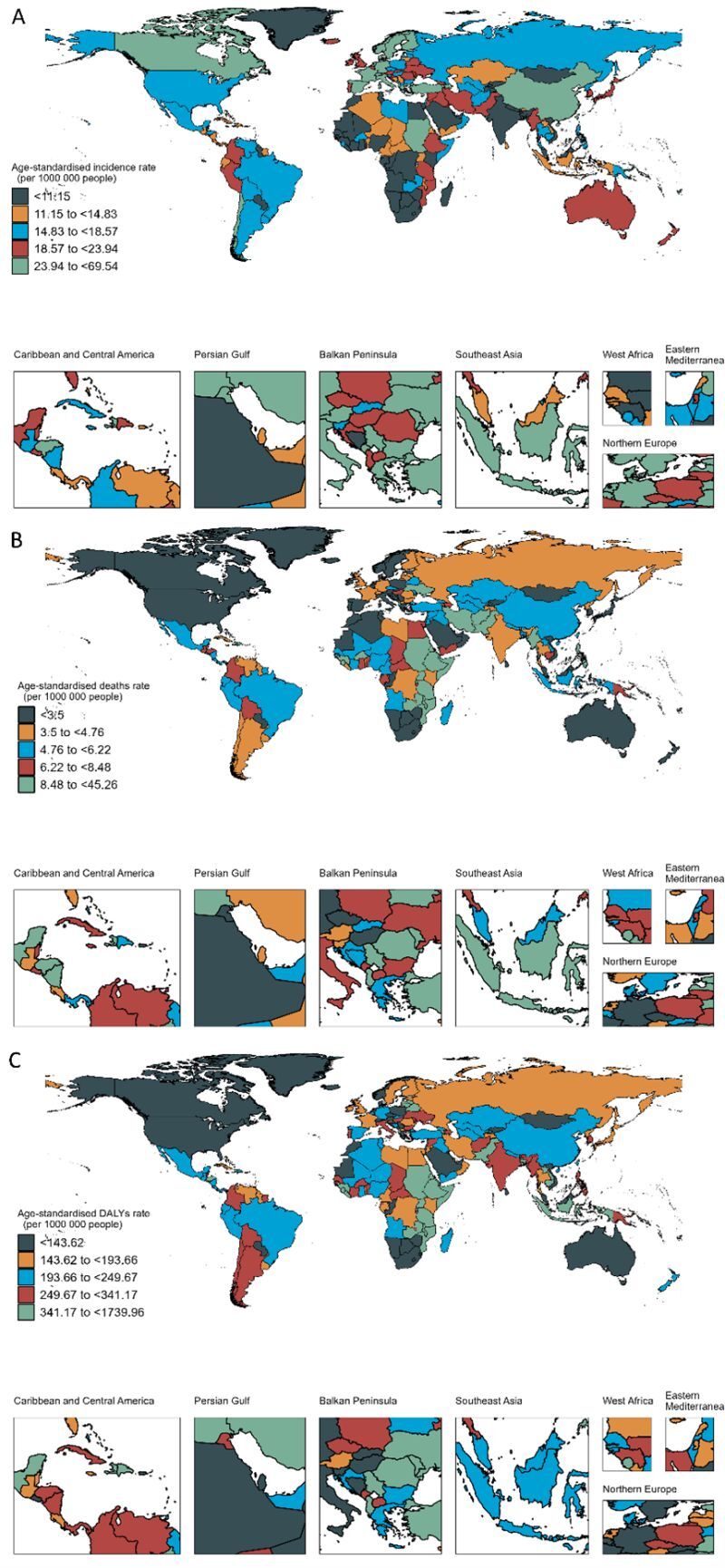
Geographical distribution of childhood cancer for both sexes combined in 2019. **Panel A.** Incidence rates (/10^5^). **Panel B.** Death rates (/10^5^). **Panel C.** DALYs rates (/10^5^). Data source: Global Burden of Diseases, Injuries, and Risk Factor Study 2019. DALYs – disability-adjusted life years, /10^5^ – per 10 000 population

At the country level, the highest incidence rates of childhood cancer were in Monaco, followed by the Democratic People’s Republic of Korea and Ethiopia (**Figure 3**, panel A). Malawi, Ethiopia, and Haiti were the three countries with the highest revealed mortality and DALY rates (**Figure 3**, panels B–C). More than 100 countries have shown an over 50% decrease in DALY burden for paediatric cancers from 1990 to 2019. Serbia showed the highest decrease in DALYs rates of paediatric cancers, whereas Burkina Faso had the most significant increases in DALYs rates from 1990 to 2019 (Figure S4 in the **Online Supplementary Document**).

### Paediatric cancer burden by SDI

**Figure 4:**
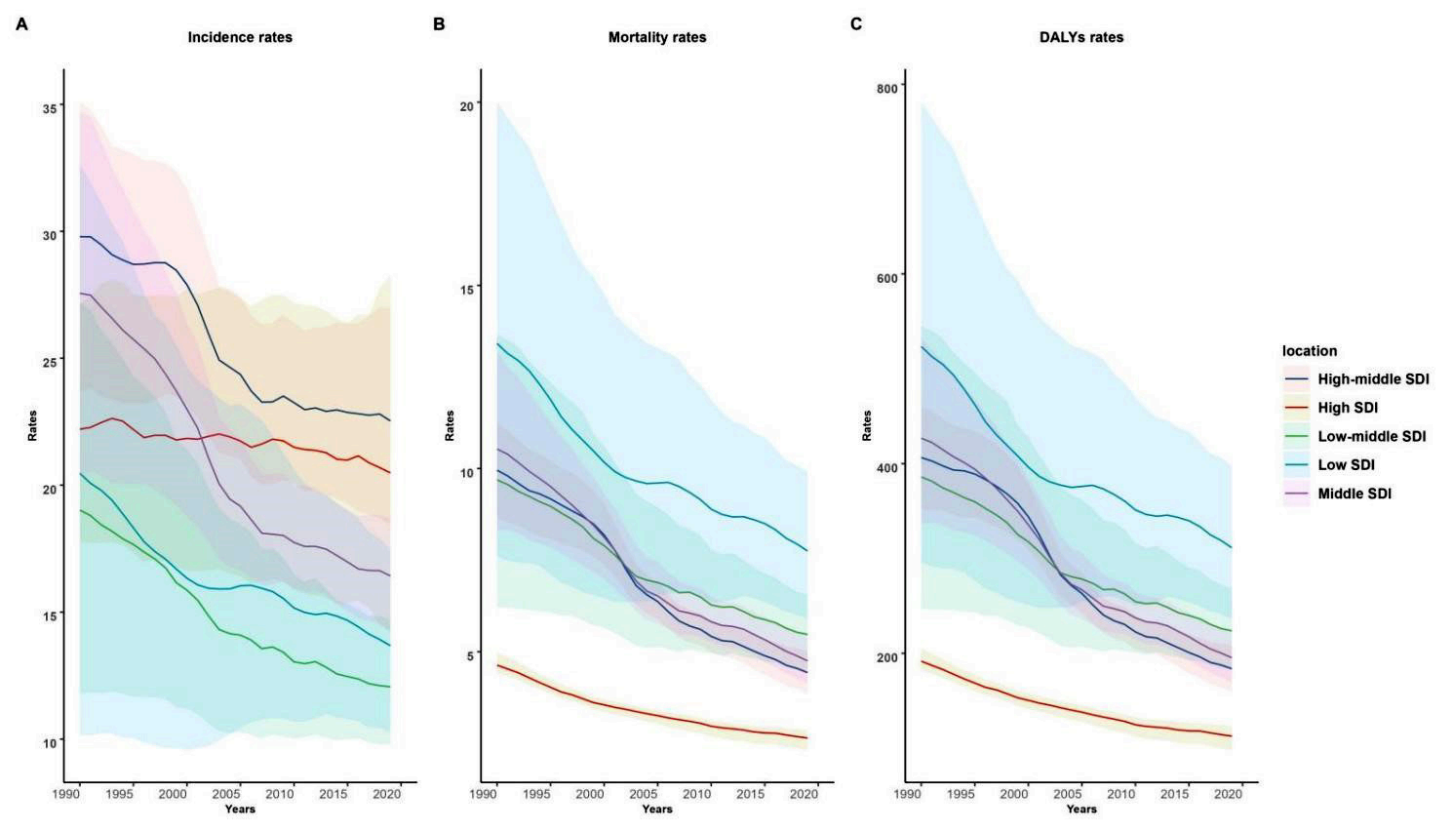
The trend in total cancer among children aged zero to nine years in both sexes from 1990 to 2019. **Panel A.** Incidence rates. **Panel B.** Mortality rates. **Panel C.** DALYs rates. DALYs – disability-adjusted life years

The burden of paediatric cancers was related to the SDI. As shown in **Table 1**, cancer incidence rates in children were more notable in regions with higher SDI, but the incidence cases were more concentrated in areas with lower SDI (**Figure 4**, panel A). In terms of the DALYs rates, most of the global burden of paediatric cancer existed in the low, low-middle, and middle SDI countries. Since 1990, the incidence, mortality, and DALYs rates of paediatric cancer have decreased in all SDI countries, especially the middle SDI countries, which showed the most remarkable decrease in incidence, mortality, and DALYs rates; however, the high SDI countries showed the lowest decrease, particularly in incidence rates (**Figure 4**, panels B–C).

For cancer in children among different age groups, we found that the highest incidence cases were in middle SDI regions in both sexes among children aged zero to four and five to nine years and in the low SDI regions among infants aged <28 days and 28–365 days. The higher mortality and DALY cases were in the low, low-middle, and middle SDI regions among children aged one to four years. However, for children aged five to nine years, the mortality and DALY numbers were higher in the middle SDI regions than in children aged zero to four years (Figure S5 in the **Online Supplementary Document**).

### Paediatric cancer burden by age group

Of the different age groups among children with cancer, the one to four years age group had the greatest contribution to global burdens of children aged zero to nine years with cancer and the absolute DALYs burden (**Figure 1**, Figure S7 in the **Online Supplementary Document**). Over the past 30 years, the main cancer burdens for the one to four years age groups were kidney, nasopharynx, and CNS cancers. The trend of these disease burdens gradually decreased globally and in many SDI regions. Still, in the low-SDI regions, the change trends were not significant or stable (Figure S8 in the **Online Supplementary Document**). For children aged zero to nine years, the main burden of cancer was attributed to leukaemia, CNS cancer, and nasopharynx cancer. Between 1990–95, there was an increase in cancer burden and a subsequent decrease in global, high-middle, and middle SDI regions. However, the results showed that the trend changed gradually and increased in low-SDI regions, especially for other malignant neoplasms (Figure S9 in the **Online Supplementary Document**). For the age group under one year old, there were large differences in cancer burden. For children aged <28 days, the leading cancers were liver cancer, HL, and lip and oral cavity cancers. The trend in cancer burden was similar to that observed in children aged one to four years (Figure S10 in the **Online Supplementary Document**). For the age group of 28–365 days old, the results revealed that the main cancers were HL, liver cancer, and CNS cancers (Figure S11 in the **Online Supplementary Document**).

The second aim was to analyse the variation in outcomes according to sex and age by calculating the global age-specific incidence, mortality, and DALYs proportion for different types of cancer. We analysed the global geographical distribution of cancer in children aged zero to nine years for incidence, mortality, DALY rates, changes from 1990 to 2019, and the most common type of cancer. The GBD 2019 study also calculated each country’s SDI, a comprehensive indicator of the social and economic conditions that impact health outcomes in each location [16]. The SDI is calculated as the geometric mean of the index (zero to one) of the total fertility rate for people younger than 25 years and the mean number of years of education for people older than 15. Zero indicates the lowest per capita income, the highest fertility rate, and the fewest years of education. The SDI has five levels – low, low-middle, middle, high-middle, and high. The GBD SDI is a comprehensive measure of income per person and has been shown to correlate well with health outcomes. In 2019, we examined the relationship between the SDI and the incidence, mortality, and DALY rates of children with cancer in different age groups.

### Cancer-specific burden among children

In 2019, leukaemia was the most common cancer, and other malignant neoplasms (uncategorised malignant neoplasms) were the most common cause of mortality and DALYs among children aged zero to nine years in both males and females. The other three common causes of death in children with cancer aged zero to nine years were brain and central nervous system cancers, acute lymphoid leukaemia, and other leukaemia.

Rankings of the relative burden of childhood cancers are shown in Figure S6 in the **Online Supplementary Document**, indicated in DALYs numbers by SDI regions, GBD super regions, and the 47 countries with the highest population of children aged zero to nine years in 2019. The inter-category rankings revealed that low-middle SDI countries had the greatest DALY burden among most cancer types in children. Sub-Saharan Africa and South Asia had the greatest burden for most childhood cancer types, and India, China, Pakistan, and Nigeria had a significantly large DALY burden for the majority of childhood cancer types in 2019. The intra-category ranking demonstrated that for most SDI regions, super GBD regions, and countries, brain and central nervous system cancer other than malignant neoplasms had the highest DALY burden among all paediatric cancer types. In the last 30 years, other leukaemia showed the most significant decrease in incidence, mortality, and DALY rates, whereas ovarian cancer showed the largest increase in incidence, mortality, and DALY rates in females. Thyroid cancer showed the most remarkable increase in incidence rates in males (**Table 1**, **Table 2**).

**Table 2:** Incidence, deaths, and DALYs of various cancers in the world regions in 2019, with 95% UI in both sexes

	Incidence			Deaths			DALYs		
**Cancer type**	**N (95% UI)**	**Rate per 10 000 population**	**Change in rates, % (95% UI)**	**N (95% UI)**	**Rate per 10 000 population**	**Change in rates, % (95% UI)**	**N (95% UI)**	**Rate per 10 000 population**	**Change in rates, % (95% UI)**
ALL	26 612 (21 560, 31 334)	2.02 (1.64, 2.38)	–0.06 (–0.41, 0.42)	11 373 (8892, 13 868)	0.86 (0.67, 1.05)	–0.38 (–0.61, –0.03)	972 810 (756 858, 1 188 398)	35.05 (27.27, 42.82)	–0.36 (–0.61, –0.01)
AML	9576 (7762, 11 797)	0.73 (0.59, 0.90)	–0.14 (–0.44, 0.32)	5272 (4301, 6446)	0.40 (0.33, 0.49)	–0.23 (–0.51, 0.20)	451 602 (367 862, 553 347)	16.27 (13.26, 19.94)	–0.21 (–0.50. 0.23)
Brain and CNS cancer	30 319 (23 232, 36 055)	2.30 (1.76, 2.74)	–0.16 (–0.57, 0.31)	15 038 (11 518, 17 986)	1.14 (0.87, 1.37)	–0.35 (–0.65, 0.04)	1 284 451 (982 095, 1 540 740)	46.28 (35.39, 55.52)	–0.34 (–0.64, 0.06)
HL	2413 (1846, 2894)	0.18 (0.14, 0.22)	–0.18 (–0.38, 0.35)	864 (590, 1112)	0.07 (0.04, 0.08)	–0.44 (–0.59, –0.02)	72 344 (49 187, 92 857)	2.61 (1.77, 3.35)	–0.43 (–0.58, 0.00)
Kidney cancer	10 994 (9345, 12 760)	0.83 (0.71, 0.97)	–0.02 (–0.23, 0.26)	2425 (1971, 2944)	0.18 (0.15, 0.22)	–0.25 (–0.4, –0.01)	211 997 (172 159, 256 837)	7.64 (6.20, 9.25)	–0.23 (–0.39, 0.02)
Lip and oral cavity cancer	459 (381, 554)	0.03 (0.03, 0.04)	0.09 (–0.09, 0.36)	134 (110, 163)	0.01 (0.01, 0.01)	–0.06 (–0.25, 0.20)	11 143 (9170, 13 525)	0.40 (0.33, 0.49)	–0.04 (–0.24, 0.23)
Liver cancer	2203 (1621, 2887)	0.17 (0.12, 0.22)	–0.35 (–0.53, –0.16)	2358 (1825, 2950)	0.18 (0.14, 0.22)	–0.43 (–0.61, –0.23)	203 000 (156 652, 254 128)	7.31 (5.64, 9.16)	–0.42 (–0.6, –0.21)
Malignant skin melanoma	1424 (771, 2943)	0.11 (0.06, 0.22)	0.24 (–0.04, 0.92)	213 (140, 366)	0.02 (0.01, 0.03)	–0.20 (–0.43, 0.25)	18 740 (12 174, 32 023)	0.68 (0.44, 1.15)	–0.18 (–0.42, 0.29)
Nasopharynx cancer	416 (366, 471)	0.03 (0.03, 0.04)	–0.28 (–0.42, –0.10)	145 (123, 168)	0.01 (0.01, 0.01)	–0.55 (–0.65, –0.42)	12 027 (10 199, 13 949)	0.43 (0.37, 0.5)	–0.54 (–0.64, –0.40)
Non-Hodgkin lymphoma	7881 (6243, 10 269)	0.60 (0.47, 0.78)	–0.07 (–0.27, 0.25)	3989 (3395, 4649)	0.30 (0.26, 0.35)	–0.41 (–0.56, –0.09)	338 398 (287 749, 394 422)	12.19 (10.37, 14.21)	–0.39 (–0.55, –0.07)
Other leukaemia	49 306 (40 493, 60 596)	3.74 (3.07, 4.6)	–0.66 (–0.77, –0.35)	7796 (6182, 9863)	0.59 (0.47, 0.75)	–0.75 (–0.83, –0.52)	696 141 (550 086, 883 845)	25.08 (19.82, 31.85)	–0.75 (–0.83, –0.50)
Other malignant neoplasms	41 469 (35 824, 48 161)	3.15 (2.72, 3.66)	–0.09 (–0.22, 0.09)	20 089 (16 293, 24 504)	1.52 (1.24, 1.86)	–0.33 (–0.46, –0.16)	1 728 045 (1 402 587, 2 111 368)	62.27 (50.54, 76.08)	–0.32 (–0.45, –0.15)
Ovarian cancer	989 (788, 1227)	0.08 (0.06, 0.09)	0.55 (–0.18, 1.26)	201 (157, 257)	0.02 (0.01, 0.02)	0.30 (–0.36, 1.00)	16 896 (13 282, 21 588)	0.61 (0.48, 0.78)	0.33 (–0.34, 1.05)
Thyroid cancer	1146 (967, 1336)	0.09 (0.07, 0.10)	0.29 (0.05, 0.65)	87 (73, 102)	0.01 (0.01, 0.01)	–0.21 (–0.36, 0.02)	7737 (6408, 9016)	0.28 (0.23, 0.32)	–0.17 (–0.32, 0.07)

ALL – acute lymphoid leukaemia, AML – acute myeloid leukaemia, CNS – central nervous system, DALY – disability-adjusted life years, HL – Hodgkin lymphoma, UI – uncertainty interval

### Brain and CNS cancer

The DALYs number trends for cancer-specific from 1990 to 2019 in different SDI regions were shown in Figures S13–14 in the **Online Supplementary Document**. The results revealed that the DALYs number trends were almost increasing in the low SDI regions except for chronic myeloid leukaemia and other leukaemia. The DALYs number trends significantly decreased in the middle SDI regions, especially for liver, kidney, and other leukaemia. AAPC and APC changes for specific cancer among one to four years and five to nine years age groups were shown in Figure S15 in the **Online Supplementary Document**. The most significant decrease from 1990 to 2019 was shown in the HL among the age group of one to four years (AAPC = –3.58) and in the leukaemia among the five to nine years age group (AAPC = –2.3). Furthermore, there was a significant decrease in DALY rates for liver cancer from 1996 to 2004 in the age group of one to four years (APC = –5.92), while the five to nine years age group had the most significant decrease in leukaemia from 1998 to 2003 (APC = –4.3).

In 2019, 30 319 (95% UI = 23 232, 36 055) incidence cases, 15 038 (95% UI = 11 518, 17 986) mortality cases, and 1 284 451 (95% UI = 982 095, 1 540 740) DALYs cases were reported worldwide among brain and CNS cancers (**Table 2**). Compared with females, males were more likely to develop brain and CNS cancer with a higher incidence, mortality, and DALYs. Among the different age groups, the mortality and DALYs of brain and CNS cancer were highest among patients aged five to nine years. However, the incidence of brain and CNS cancer was the lowest among children aged <28 days (**Figure 1**). Our analysis showed that between 1990–2019, the highest decrease in DALYs rate was observed in the one to four years age group (AAPC = –1.74), while there was an increasing change in brain and CNS cancer between 1990–98 in the five to nine years age group (APC = 0.06) (Figure S15 in the **Online Supplementary Document**). Among all cancer types in children, brain and CNS cancer had the highest DALYs burden in the high, high-middle, and middle SDI regions in 2019 (**Figure 4**). The DALYs number trend of brain and CNS cancer from 1990 to 2019 generally decreased, especially in the middle SDI regions. Still, the trend increased in the low SDI regions (Figure S13 in the **Online Supplementary Document**).

Among the GBD super regions, East Asia had the highest incidence rates of brain and CNS cancer in 2019, while Tropical Latin America and Central Asia had the highest mortality and DALY rates (**Table 3**). The brain and CNS cancer burden showed the most significant decrease in incidence rates in the Caribbean, while Central sub-Saharan Africa showed the largest decrease from 1990 to 2019. However, East Asia showed the largest decrease in DALY rates for brain and CNS cancers from 1990 to 2019 (**Table 3**). Furthermore, the DALYs rates of brain and CNS cancer in 2019 had one of the highest rates in Albania, Haiti, and Armenia, where the DALYs rates were all more than 90 for 100 000 persons (Figure S12 in the **Online Supplementary Document**).

**Table 3:** Worldwide incidence, deaths, DALYs, and rates by SDI and regions for brain and CNS cancer in 2019 in children younger than 10 y and the change in trends from 1990 to 2019 in both sexes

	Incidence					Deaths					DALYs						
**Location**	**N (95% UI)**	**Rate per 10 000 population**		**Change in rates, % (95% UI)**		**N (95% UI)**		**Rate per 10 000 population**	**Change in rates, % (95% UI)**		**N (95% UI)**		**Rate per 10 000 population**		**Change in rates, % (95% UI)**		
Global	30 319 (23 232, 36055)	2.30 (1.76, 2.74)		–0.16 (–0.57, 0.31)		15 038 (11 518, 17 986)		1.14 (0.87, 1.37)	–0.35 (–0.65, 0.04)		1 284 451 (982 095, 1 540 740)		46.28 (35.39, 55.52)		–0.34 (–0.64, 0.06)		
SDI																	
*High*	3295 (2285, 3976)	3.08 (2.14, 3.72)		0.05 (–0.43, –0.30)		907 (636, 1021)		0.85 (0.59, 0.95)	–0.26 (–0.61, –0.15)		77 706(54 396, 87 549)		34.98 (24.49, 39.41)		–0.25 (–0.6, –0.13)		
*High-middle*	5230 (3462, 6309)	3.18 (2.10, 3.84)		0.00 (–0.51, 0.43)		2018 (1366, 2376)		1.23 (0.83, 1.44)	–0.38 (–0.69, –0.13)		172 276 (116 551, 203 311)		49.86 (33.73, 58.85)		–0.37 (–0.69, –0.12)		
*Middle*	8645 (6337, 10 516)	2.34 (1.72, 2.85)		–0.20 (–0.63, 0.39)		4051 (2966, 4906)		1.10 (0.80, 1.33)	–0.41 (–0.71, 0.01)		344 598 (251 808, 418 090)		44.57 (32.57, 54.07)		–0.4 (–0.71, 0.04)		
*Low-middle*	7175 (5511, 8934)	2.07 (1.59, 2.58)		–0.23 (–0.62, 0.45)		4230 (3291, 5255)		1.22 (0.95, 1.52)	–0.33 (–0.67, 0.29)		361 225 (280 835, 449 436)		49.77 (38.69, 61.92)		–0.31 (–0.66, 0.33)		
*Low*	5955 (4263, 8178)	1.81 (1.30, 2.49)		–0.27 (–0.60, 0.42)		3821 (2724, 5287)		1.16 (0.83, 1.61)	–0.24 (–0.59, 0.48)		327 776 (233 035, 454 202)		46.38 (32.97, 64.27)		–0.22 (–0.58, 0.53)		
Africa																	
*Central sub-Saharan*	345 (195, 640)	0.86 (0.49, 1.60)		–0.43 (–0.69, 0.45)		241 (136, 442)		0.60 (0.34, 1.10)	–0.43 (–0.69, 0.46)		20 437 (11 541, 37 767)		23.83 (13.46, 44.03)		–0.40 (–0.67, 0.53)		
*Eastern sub-Saharan*	1954 (1429, 2751)	1.59 (1.16, 2.24)		–0.22 (–0.64, 0.77)		1332 (976, 1859)		1.08 (0.79, 1.51)	–0.23 (–0.64, 0.77)		114 609 (83 837, 159 582)		43.3 (31.67, 60.29)		–0.21 (–0.63, 0.82)		
*North and Middle East*	3437 (2358, 4324)	2.88 (1.98, 3.63)		–0.05 (–0.54, 0.80)		1446 (1011, 1799)		1.21 (0.85, 1.51)	–0.32 (–0.64, 0.26)		122 939 (85 731, 153 358)		49.06 (34.21, 61.2)		–0.3 (–0.63, 0.31)		
*Southern sub-Saharan*	124 (86, 158)	0.77 (0.54, 0.98)		0.08 (–0.41, 0.63)		79 (56, 102)		0.49 (0.35, 0.63)	0.02 (–0.43, 0.54)		6676 (4675, 8564)		19.69 (13.78, 25.25)		0.02 (–0.43, 0.55)		
*Western sub-Saharan*	1942 (1139, 3252)	1.40 (0.82, 2.34)		0.11 (–0.40, 0.88)		1305 (790, 2190)		0.94 (0.57, 1.58)	0.09 (–0.42, 0.9)		112 075 (67 590, 188 643)		37.37 (22.54, 62.9)		0.13 (–0.4, 0.96)		
America																	
*Andean Latin*	260 (157, 355)	2.10 (1.27, 2.86)		–0.01 (–0.68, 0.89)		155 (92, 210)		1.25 (0.74, 1.7)	–0.13 (–0.72, 0.63)		13 057 (7769, 17 760)		49.55 (29.48, 67.4)		–0.12 (–0.71, 0.66)		
*Central Latin*	729 (414, 983)	1.68 (0.95, 2.27)		0.11 (–0.58, 0.60)		395 (223, 521)		0.9 1(0.51, 1.20)	–0.02 (–0.62, 0.41)		33 417 (18 837, 44 181)		36.72 (20.70, 48.55)		0.01 (–0.61, 0.45)		
*High-income North*	1372 (991, 1710)	3.21 (2.32, 4.00)		0.04 (–0.32, 0.33)		388 (295, 436)		0.91 (0.69, 1.02)	–0.18 (–0.47, –0.07)		33 197 (25 135, 37 399)		37.34 (28.27, 42.07)		–0.16 (–0.45, –0.05)		
*Southern Latin*	203 (155, 257)	2.04 (1.55, 2.58)		0.01 (–0.44, 0.50)		103 (82, 125)		1.04 (0.82, 1.25)	–0.14 (–0.52, 0.22)		8745 (6948, 10 552)		42.38 (33.67, 51.14)		–0.12 (–0.51, 0.24)		
*Tropical Latin*	1142 (660, 1479)	3.49 (2.02, 4.52)		0.06 (–0.61, 0.61)		594 (352, 765)		1.82 (1.07, 2.34)	–0.08 (–0.65, 0.41)		50 461 (29 808, 65 085)		74.01 (43.72, 95.45)		–0.09 (–0.66, 0.40)		
*Caribbean*	174 (82, 332)	2.21 (1.05, 4.22)		0.67 (–0.33, 2.05)		111 (50, 221)		1.41 (0.64, 2.8)	0.61 (–0.35, 1.93)		9439 (4267, 18 794)		56.97 (25.75, 113.43)		0.65 (–0.33, 2.02)		
Asia																	
*Central*	434 (331, 537)	2.30 (1.75, 2.84)		0.1 (–0.51, 0.82)		276 (211, 341)		1.46 (1.12, 1.81)	0.04 (–0.53, 0.71)		23 311 (17 823, 28 782)		58.41 (44.66, 72.12)		0.07 (–0.52, 0.76)		
*East*	6231 (4306, 8168)	3.91 (2.70, 5.12)		–0.08 (–0.58, 0.88)		2213 (1567, 2913)		1.39 (0.98, 1.83)	–0.51 (–0.77, 0.00)		189 670 (134 474, 249 747)		55.26 (39.18, 72.76)		–0.51 (–0.77, 0.01)		
*High-income Pacific*	501 (331, 620)	3.29 (2.17, 4.07)		0.21 (–0.52, 0.67)		99 (66, 114)		0.65 (0.43, 0.75)	–0.29 (–0.73, –0.10)		8583 (5692, 9877)		27.51 (18.25, 31.66)		–0.29 (–0.73, –0.10)		
*South*	7633 (5525, 9898)	2.26 (1.64, 2.94)		–0.15 (–0.58, 0.7)		4407 (325, 5607)		1.31 (0.96, 1.66)	–0.29 (–0.65, 0.43)		377 383 (278 074, 479 990)		54.04 (39.82, 68.73)		–0.27 (–0.64, 0.47)		
*Southeast*	1463 (1075, 1852)	1.31 (0.97, 1.67)		–0.22 (–0.60, 0.48)		940 (685, 1200)		0.84 (0.62, 1.08)	–0.28 (–0.63, 0.40)		79 342 (57 878, 101 309)		34.35 (25.06, 43.86)		–0.26 (–0.62, 0.42)		
Europe																	
*Central*	276 (209, 354)	2.39 (1.81, 3.06)		–0.34 (–0.66, –0.08)		141 (105, 177)		1.22 (0.91, 1.53)	–0.47 (–0.73, –0.26)		11 958 (8941, 15 037)		49.92 (37.32, 62.77)		–0.48 (–0.73, –0.27)		
*Eastern*	514 (364, 615)	2.02 (1.43, 2.42)		–0.22 (–0.55, 0.03)		334 (236, 400)		1.31 (0.93, 1.57)	–0.25 (–0.57, –0.03)		28 227 (19 981, 33 858)		53.92 (38.17, 64.68)		–0.25 (–0.57, –0.03)		
*Western*	1423 (908, 1764)	3.15 (2.01, 3.90)		–0.07 (–0.51, 0.19)		395 (256, 453)		0.87 (0.57, 1.00)	–0.37 (–0.66, –0.25)		33 842 (21 906, 39 015)		36.2 (23.43, 41.73)		–0.36 (–0.66, –0.24)		
Oceania																	
*Oceania*	73 (34, 154)	2.11 (0.99, 4.48)		0.11 (–0.32, 1.02)		49 (23, 103)		1.42 (0.67, 3.00)	0.11 (–0.32, 1.02)		4207 (1962, 8946)		55.88 (26.06, 118.83)		0.11 (–0.33, 1.02)		
*Australasia*	90 (60, 118)	2.46 (1.64, 3.24)		–0.18 (–0.56, 0.11)		34 (23, 41)		0.92 (0.64, 1.13)	–0.3 (–0.62, –0.13)		2877 (1984, 3513)		37.55 (25.9, 45.87)		–0.3 (–0.62, –0.12)		

CNS – central nervous system, DALYs – disability-adjusted life years, SDI – sociodemographic index, UI – uncertainty interval

### Leukaemia

In 2019, 26 612 (95% UI = 21 560, 31 334) incidence cases, 11 373 (95% UI = 8 892, 13 868) mortality cases, and 972 810 (95% UI = 756 858, 1 188 398) DALYs cases were recorded for ALL worldwide (**Table 2**). The incidence, mortality, and DALYs rates of ALL were slightly higher in males than females (Tables S4–5 in the **Online Supplementary Document**). Regarding the age groups, the mortality and DALYs cases of ALL were the highest among children aged five to nine years. ALL had higher proportions among children aged 28–365 days and five to nine years than in other age groups (**Figure 1**). In 2019, the highest DALYs cases of ALL were observed in low-middle SDI regions, and the high SDI regions had the lowest DALYs burden of ALL. Furthermore, there was a significant decrease in the trend of ALL and AML from 1990 to 2019 in the middle and high-middle SDI regions. Still, the low SDI regions had an increasing trend (Figure S14 in the **Online Supplementary Document**). Among the 21 regions, Western Europe had the highest incidence rates in 2019, and Central Latin America had the highest mortality and DALYs rates. However, the lowest incidence, mortality, and DALYs rates of ALL occurred in Southern sub-Saharan Africa. Over the past 30 years, East Asia has had the largest increase in ALL incidence, while Central sub-Saharan Africa has shown the most significant decrease. The mortality and DALYs changes of ALL have decreased in all regions, and Eastern Europe had the highest decreases for the ALL (**Table 4**). The highest DALY rates of ALL in 2019 were in Ethiopia and Haiti, where the rates were more than 120 per 100 000 persons (Figure S12 in the **Online Supplementary Document**).

**Table 4:** Worldwide incidence, deaths, DALYs, and rates by SDI and regions for ALL in 2019 in children younger than 10 y and the change in trends from 1990 to 2019 in both sexes

		Incidence						Deaths						DALYs				
**Location**		**N (95% UI)**		**Rate per 10 000 population**		**Change in rates, % (95% UI)**		**N (95% UI)**		**Rate per 10 000 population**		**Change in rates, % (95% UI)**		**N (95% UI)**		**Rate per 10 000 population**		**Change in rates, % (95% UI)**
Global		328 (206, 468)		2.64 (1.66, 3.78)		–0.09 (–0.59, 0.58)		328 (206, 468)		2.64 (1.66, 3.78)		–0.09 (–0.59, 0.58)		328 (206, 468)		2.64 (1.66, 3.78)		–0.09 (–0.59, 0.58)
SDI																			
*High*		3606 (3012, 4379)		3.37 (2.81, 4.09)		0.22 (–0.05, 0.61)		334 (296, 382)		0.31 (0.28, 0.36)		–0.57 (–0.64, –0.45)		30 324 (26 560, 35 014)		13.65 (11.96, 15.76)		–0.54 (–0.62, –0.41)
*High-middle*		6139 (4815, 7679)		3.73 (2.93, 4.67)		0.48 (0.010, 1.05)		1307 (1064, 1548)		0.79 (0.65, 0.94)		–0.48 (–0.64, –0.31)		113 609 (92 084, 134 313)		32.88 (26.65, 38.87)		–0.46 (–0.63, –0.28)
*Middle*		7306 (5701, 8877)		1.98 (1.54, 2.40)		–0.03 (–0.42, 0.58)		3276 (2609, 3908)		0.89 (0.71, 1.06)		–0.37(–0.61, 0.01)		278 723 (221 631, 333 015)		36.05 (28.66, 43.07)		–0.36 (–0.60, 0.03)
*Low-middle*		4251 (3357, 5539)		1.23 (0.97, 1.60)		–0.34 (–0.62, 0.14)		2813 (2236, 3585)		0.81 (0.64, 1.03)		–0.39 (–0.65, 0.09)		237 733 (188 757, 303 274)		32.75 (26.01, 41.78)		–0.37 (–0.65, 0.13)
*Low*		5293 (3385, 7432)		1.61 (1.03, 2.26)		–0.38 (–0.70, 0.34)		3633 (2323, 5048)		1.10 (0.71, 1.53)		–0.35 (–0.70, 0.45)		311 601 (198 505, 434 530)		44.09 (28.09, 61.49)		–0.33 (–0.69, 0.49)
Africa																			
*Central sub-Saharan*		252 (131, 469)		0.63 (0.33, 1.17)		–0.41 (–0.72, 0.72)		181 (94, 336)		0.45 (0.23, 0.84)		–0.40 (–0.72, 0.77)		15 353 (7956, 28 527)		17.90 (9.28, 33.26)		–0.38 (–0.71, 0.85)
*Eastern sub-Saharan*		2960 (1428, 4584)		2.41 (1.16, 3.73)		–0.37 (–0.74, 0.64)		2055 (1000, 3118)		1.67 (0.81, 2.54)		–0.37 (–0.74, 0.59)		177 834 (86 085, 271 234)		67.18 (32.52, 102.47)		–0.35 (–0.73, 0.64)
*North and Middle East*		1428 (948, 1865)		1.20 (0.80, 1.56)		–0.35 (–0.64, 0.22)		818 (538, 1076)		0.69 (0.45, 0.90)		–0.48 (–0.70, –0.04)		68 987 (45 304, 91 083)		27.53 (18.08, 36.35)		–0.46 (–0.69, –0.01)
*Southern sub-Saharan*		64 (42, 84)		0.40 (0.26, 0.52)		–0.09 (–0.47, 0.56)		44 (29, 58)		0.27 (0.18, 0.36)		–0.14 (–0.49, 0.49)		3672 (2427, 4832)		10.83 (7.16, 14.25)		–0.14 (–0.49, 0.51)
*Western sub-Saharan*		1157 (770, 1669)		0.83 (0.55, 1.20)		–0.16 (–0.43, 0.38)		794 (525, 1138)		0.57 (0.38, 0.82)		–0.17 (–0.44, 0.40)		68 283 (44 916, 98 346)		22.77 (14.98, 32.79)		–0.14 (–0.42, 0.44)
America																			
*Andean Latin*		328 (206, 468)		2.64 (1.66, 3.78)		–0.09 (–0.59, 0.58)		213 (135, 300)		1.72 (1.09, 2.42)		–0.17 (–0.62, 0.44)		17 967 (11 389, 25 403)		68.19 (43.22, 96.41)		–0.16 (–0.62, 0.45)
*Central Latin*		1324 (1016, 1676)		3.05 (2.34, 3.86)		–0.06 (–0.32, 0.24)		748 (585, 924)		1.73 (1.35, 2.13)		–0.20 (–0.41, 0.04)		63 334 (49 324, 78 293)		69.59 (54.2, 86.03)		–0.18 (–0.40, 0.07)
*High-income North*		603 (475, 764)		1.41 (1.11, 1.79)		–0.17 (–0.37, 0.15)		134 (121, 159)		0.31 (0.28, 0.37)		–0.51 (–0.57, –0.37)		11 574 (10 410, 13 671)		13.02 (11.71, 15.38)		–0.49 (–0.56, –0.35)
*Southern Latin*		204 (159, 260)		2.04 (1.59, 2.61)		–0.08 (–0.31, 0.20)		97 (81, 117)		0.97 (0.82, 1.17)		–0.37 (–0.49, –0.22)		8205 (6871, 9834)		39.76 (33.3, 47.66)		–0.35 (–0.48, –0.20)
*Tropical Latin*		670 (529, 828)		2.05 (1.62, 2.53)		–0.29 (–0.49, –0.06)		385 (304, 474)		1.18 (0.93, 1.45)		–0.38 (–0.56, –0.17)		32 554 (25 733, 40 140)		47.74 (37.74, 58.87)		–0.38 (–0.56, –0.18)
*Caribbean*		206 (121, 328)		2.61 (1.54, 4.17)		–0.05 (–0.39, 0.41)		135 (76, 224)		1.72 (0.96, 2.84)		–0.08 (–0.43, 0.38)		11 443 (6394, 18 991)		69.07 (38.59, 114.62)		–0.06 (–0.41, 0.42)
Asia																			
*Central*		247 (186, 320)		1.31 (0.98, 1.69)		–0.39 (–0.56, –0.17)		156 (117, 202)		0.83 (0.62, 1.07)		–0.45 (–0.6, –0.24)		13 154 (9904, 16 965)		32.96 (24.82, 42.51)		–0.44 (–0.59, –0.22)
*East*		7326 (4945, 9641)		4.60 (3.10, 6.05)		1.52 (0.08, 4.19)		1556 (1060, 1933)		0.98 (0.67, 1.21)		–0.30 (–0.69, 0.43)		136 115 (92 641, 168 915)		39.65 (26.99, 49.21)		–0.28 (–0.69, 0.47)
*High-income Pacific*		745 (558, 993)		4.89 (3.66, 6.52)		0.64 (0.05, 1.66)		48 (41, 59)		0.32 (0.27, 0.39)		–0.61 (–0.70, –0.40)		4540 (3832, 5597)		14.55 (12.28, 17.94)		–0.58 (–0.68, –0.36)
*South*		3860 (2934, 5196)		1.15 (0.87, 1.54)		–0.35 (–0.63, 0.19)		2528 (1951, 3337)		0.75 (0.58, 0.99)		–0.42 (–0.68, 0.09)		212 475 (163 261, 281 022)		30.42 (23.38, 40.24)		–0.40 (–0.67, 0.13)
*Southeast*		1489 (1135, 1912)		1.34 (1.02, 1.72)		–0.26 (–0.56, 0.40)		1042 (779, 1337)		0.94 (0.70, 1.20)		–0.30 (–0.59, 0.32)		88 134 (65 827, 113 312)		38.16 (28.5, 49.06)		–0.28 (–0.58, 0.35)
Europe																			
*Central*		239 (185, 304)		2.06 (1.60, 2.63)		0.03 (–0.25, 0.36)		53 (44, 63)		0.46 (0.38, 0.54)		–0.63 (–0.71, –0.54)		4568 (3830, 5458)		19.07 (15.99, 22.78)		–0.62 (–0.71, –0.53)
*Eastern*		612 (505, 733)		2.41 (1.99, 2.88)		–0.41 (–0.55, –0.26)		209 (175, 246)		0.82 (0.69, 0.97)		–0.67 (–0.75, –0.59)		17 887 (15 001, 21 138)		34.17 (28.65, 40.38)		–0.66 (–0.75, –0.58)
*Western*		2744 (2225, 3361)		6.07 (4.92, 7.44)		0.22 (–0.11, 0.61)		142 (121, 164)		0.31 (0.27, 0.36)		–0.62 (–0.70, –0.49)		13 662 (11 535, 16 115)		14.61 (12.34, 17.24)		–0.57 (–0.66, –0.44)
Oceania																			
*Oceania*		31 (15, 61)		0.91 (0.45, 1.78)		–0.02 (–0.43, 0.71)		22 (11, 44)		0.65 (0.32, 1.27)		–0.03 (–0.44, 0.70)		1900 (940, 3743)		25.24 (12.48, 49.72)		–0.03 (–0.44, 0.69)
*Australasia*		123 (81, 184)		3.37 (2.21, 5.02)		0.19 (–0.29, 0.94)		13 (10, 16)		0.36 (0.28, 0.44)		–0.61 (–0.70, –0.49)		1169 (931, 1462)		15.27 (12.15, 19.09)		–0.59 (–0.68, –0.46)

ALL – acute lymphoid leukaemia, DALY – disability-adjusted life years, SDI – sociodemographic index, UI – uncertainty interval

In 2019, the incidence cases (n = 2413; 95% UI = 1846, 2894), mortality cases (n = 864; 95% UI = 590, 1112) and DALYs cases (n = 72 344; 95% UI = 49 187, 92 857) of HL were observed worldwide (**Table 2**). Notably, the higher DALYs rates were in the five to nine years age group compared to the one to four years age group. There were decreasing change trends from 1990 to 2019 in the five to nine years age group (AAPC = –1.63) and one to four years age group (AAPC = –3.58) (Figure S15 in the **Online Supplementary Document**). A similar increasing DALYs rates trend for the HL and NHL from 1990 to 2019 was observed in the low SDI regions, and there were the highest DALY rates of HL and NHL in 2019 in the low SDI regions (Figure S13 in the **Online Supplementary Document**). Our analysis showed the highest DALY rates of NHL in 2019 in the Solomon Islands and Haiti, with rates of more than 50 for 100 000 persons (Figure S12 in the **Online Supplementary Document**).

## DISCUSSION

The GBD 2019 study showed that there were 206 602 incidence cancer cases worldwide in 2019, with a mortality rate of 72 190 and DALYs of 6.2 million. Between 1990–2019, the number of new global cancer cases and deaths among children of zero to nine years decreased by 35 and 46%, respectively. The results showed ongoing epidemiologic transitions, demographic shifts, and disparities in cancer prevention, care and control [24]. Furthermore, these results provide comprehensive and comparable evaluations that can potentially demonstrate reducing the total burden of children’s cancer worldwide. Cancer is a rare cause of mortality in children compared to other diseases such as maternal and neonatal disorders, respiratory infections, tuberculosis, and enteric infections. However, children with cancer face long-term challenges in diagnosis and treatment, leading to financial losses and serious chronic complications [25]. In our analysis of the GBD 2019, we reported the absolute rates of global paediatric cancer incidences, deaths, and DALYs. In 2019, our analysis showed that the global DALYs, YLLs, and YLDs rates of total cancer among children zero to nine years were –2.04 (95% UI = –2.16, –1.92), –2.12 (95% UI = –2.24, –1.99), and –1.48 (95% UI = –1.58, –1.38) respectively. DALYs provide a useful summary measure of early mortality and treatment-related mortality, particularly for children with cancer, because early deaths lead many YLLs to DALYs, and surviving children often live with many chronic disabilities. We found widening changes in all-cause mortality due to cancer between countries, particularly among different age groups, as well as large differences in the main causes of death between different regions of the world. Compared with previous studies [3,10,13], this research showed a steady decline in the incidence, mortality, and DALYs due to paediatric cancers among children aged zero to nine years worldwide. It highlighted that an accurate estimate of paediatric cancer burden using comparable measures in different age groups and regions is essential for health policy decision-making.

We provided estimations by the quintile of SDI, which is a systematic measure to indicate educational attainment, income per capita, and total fertility rate for those younger than 25 years. Since 1990, low SDI regions have been notable for having the highest death and DALY rates; however, the high SDI regions have been stable, decreasing, and at the lowest rates. In high-SDI regions, advanced screening measures, medical care systems, and easier access to health care have led to a downward trend in deaths. The previous study was also similar to our results [10], which showed the majority of the DALY cancer burden occurring in the lower SDI regions among adolescents and young adults. Thus, there is an opportunity for governments in the lower SDI regions to effectively address the gap and suggest these countries develop an amendment specific to children aged zero to nine years by underlining the problems faced by these patients.

As expected, the highest incidence rates of total cancer in male children aged zero to nine years were in Western Europe and for females in East Asia, while the lowest incidence rates of total cancers were in Southern sub-Saharan Africa. Unequal health resource distributions resulted in inadequate health care infrastructure in lower SDI regions, which led to delayed diagnosis, delayed treatment of paediatric cancers, and children with cancers with other comorbidities such as malnutrition and infections. However, the association between paediatric cancer incidence rates and the SDI was influenced by multiple factors. Ethnicity-related genetic variations and environmental risk exposure may result in differences in the cancer burden on children between regions and nations. Recognising the strong connections between socioeconomic status and health and reducing the large contribution of cancer to the overall disease burden is the first step in prioritising policies for cancer prevention and treatment.

Globally, the array of cancer types is unique in children aged zero to nine years compared to adolescents and young adults [10]. The leading causes of cancer-related deaths among children in 2019 fell into four categories: other malignant neoplasms, brain and CNS cancer, ALL, and other leukaemia. We found general sex differences in the trends among paediatric cancers, with notably higher incidence, mortality, and DALYs and a slower extent of decline in males than in females. However, child patients often do not have a reasonable health care system and need to focus on particular treatment programmes because of age restrictions. There were differences in cancer burden in different age groups, and our study showed the leading cancer burden in different age groups. For brain and CNS cancer, the low SDI region had the highest ranking by the numbers of DALYs among children aged zero to nine years. When comparing the different age groups, we found that the brain and CNS cancers mainly contributed to the DALYs burden on children aged one to four years, particularly among five to nine-year-old children. Over the past 30 years, the trend of brain and CNS cancers has been stably decreasing in the five to nine-year-old children compared to the one to four years old. However, the global burden of brain and CNS cancer in all ages has increased over the past 25 years [24]. Brain and CNS cancer are a substantial source of mortality among paediatric cancers worldwide. To reduce the burden of brain and CNS cancer, effective multimodal treatments, such as neurosurgical care, radiation, and chemotherapy, are required. Owing to the high mortality rates and inherently disabling effects, the administration must improve the health care system and establish early detection and treatment [24].

With new treatments such as targeted inhibitors, chimeric antigen receptors, and immunotherapy, mortality due to leukaemia among children aged zero to nine years has recently significantly decreased in the higher SDI regions. However, leukaemia is still a highly prevalent disease that leads to considerable disability and increased economic costs in the low SDI regions. Our study showed that ALL, AML, and other leukaemia were the leading causes of DALY burden among children aged five to nine years, and the trend of these disease burdens among childhood cancer gradually decreased in the high SDI regions. In recent years, second-generation tyrosine kinase inhibitors and improvements in treatment schedules in regions with higher SDI have prolonged overall survival and improved prognosis in patients with leukaemia [26,27]. For infants aged 28–365 days, HL accounted for the leading cancer burden over the past 30 years. HL burden trend has declined significantly in the middle and high-middle SDI regions but is stable and high in the low SDI regions. Therefore, countries and territories with low SDI need greater investment and research to improve treatment methods and reduce the leukaemia burden among children.

The GBD study 2019 provided high-quality estimates of the global cancer burden. Compared to a previous GBD study that estimated the cancer burden among children and adolescents [6], this study provided a more specific and comprehensive analysis of the cancer burden among children aged zero to nine years. We have also provided detailed information about the cancer burden in different age groups. This study has some limitations. First, the catalogue of cancers was incomplete for children aged zero to nine years. Mortality data were derived from local registrations and statistical systems that did not cover the entire population. The best estimation source for mortality data was autopsy data. However, autopsies could not accurately indicate the causes of death. Morphology classification was important for accurately diagnosing and treating children’s cancer. There is a lack of possibility of directly comparing all the data in a consolidated data source because of countries’ different opportunities to diagnose and treat cancer. Another limitation is the racial differences. Our study estimated children’s cancer burden by age and sex but not by race. Cancer occurs in all racial groups, and some studies have demonstrated that different racial groups are affected by different cancer types [28]. Finally, our study concentrated on estimates in 2019. It did not include the direct and indirect effects of the coronavirus disease 2019 (COVID-19) pandemic on the cancer burden for children aged zero to nine. We should consider the effect of the COVID-19 pandemic on the predictions of the children’s cancer burden, and this will be a crucial area of consideration in future studies as data becomes available.

## CONCLUSIONS

Our study showed a comprehensive analysis of the distribution of childhood cancers by sex, age group, and area and its changing trend over the past 30 years worldwide. The overall cancer burden in children has been decreasing worldwide over the past 30 years; however, countries with low SDI still show a higher burden compared to the high SDI regions. Despite the decreasing trend of all cancer types burden globally, other malignant neoplasms, brain and CNS cancer, and ALL remain the most common cancers and the leading cause of death among children aged zero to nine years. Policymakers should focus on receiving more detailed information about the cancer burden, which is better for allocating resources and implementing effective measures in lower SDI regions. Our study also emphasises that future research should concentrate on advanced treatment and prevention strategies for childhood cancer, such as brain and CNS cancer and ALL.

## Additional material


Online Supplementary Document
