# Supplementary material for: Global, regional, and national burdens of cancer in children aged zero to nine years from 1990 to 2019
Source: J Glob Health. 2024 May 31;14:04104. doi: 10.7189/jogh.14.04104 (PMC11140426; doi:10.7189/jogh.14.04104)
Supplement: Online Supplementary Document [file jogh-14-04104-s001.pdf]

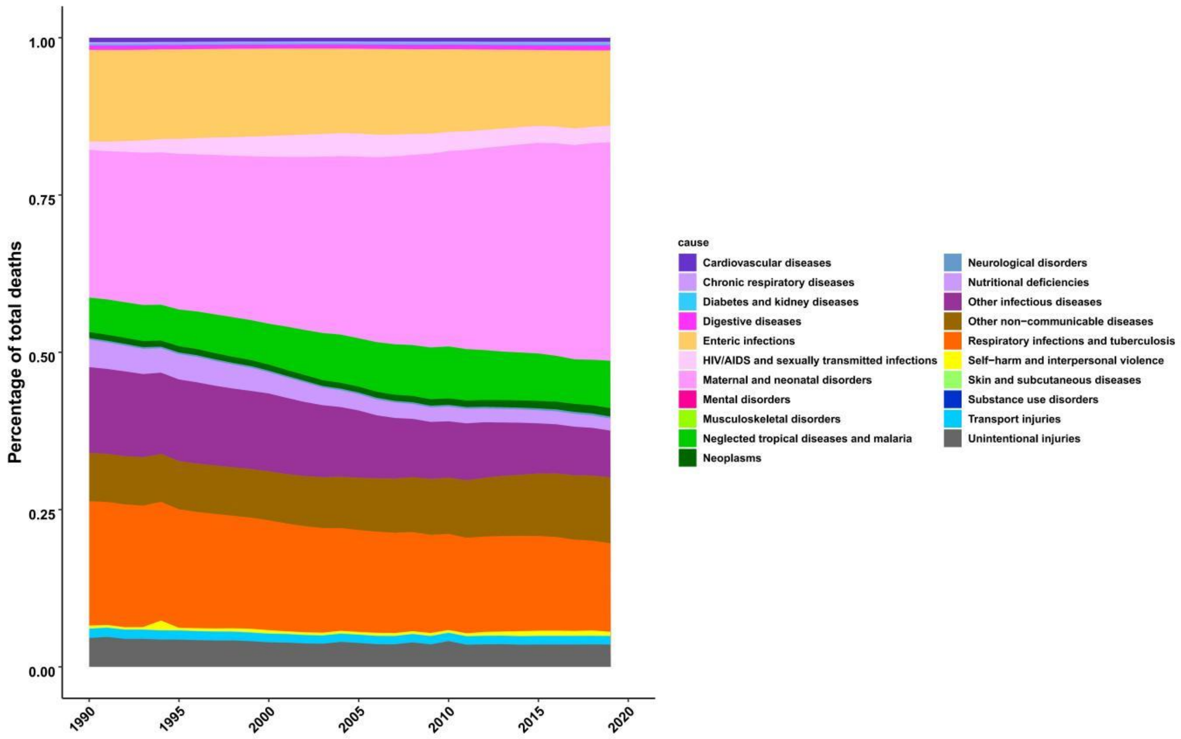

Supplementary Figure 1 Percentage of total deaths by cause in people aged 0-9 years from 1990 to 2019 in both sexes

GBD = Global Burden of Diseases, Injuries, and Risk Factors Study

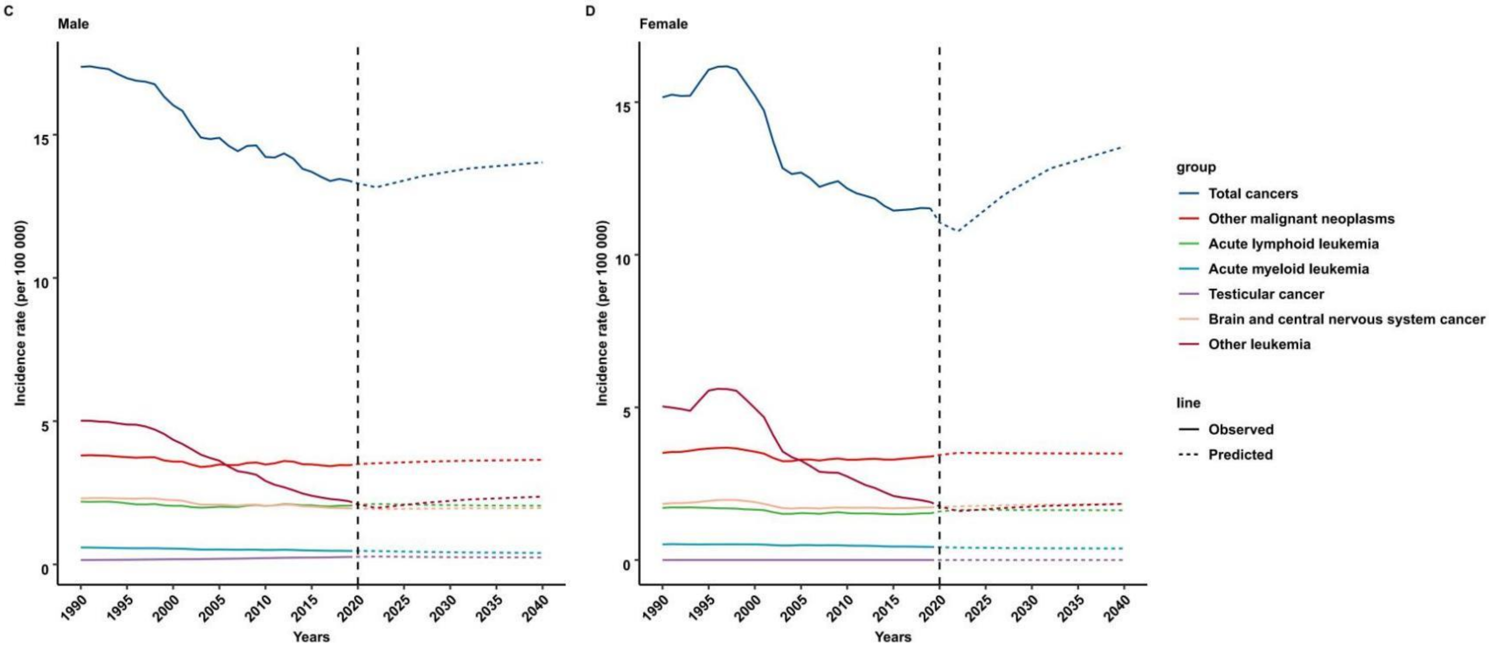

Supplementary Figure 2 Prediction of the incidence rate of common childhood cancers by sex in 2040 in A, B (0 to 4 years) and C, D (5 to 9 years)

"Other malignant neoplasms" comprise all malignancies without a separate GBD cause category listed; this category does not include non-melanoma skin cancers and myelodysplatic or myeloprolifeative neoplasms, which are separate GBD cause categories not included in this analysis.Other leukaemia include leukaemia not otherwise specified. GBD = Global Burden of Disease, Injuries, and Risk Factor Study.

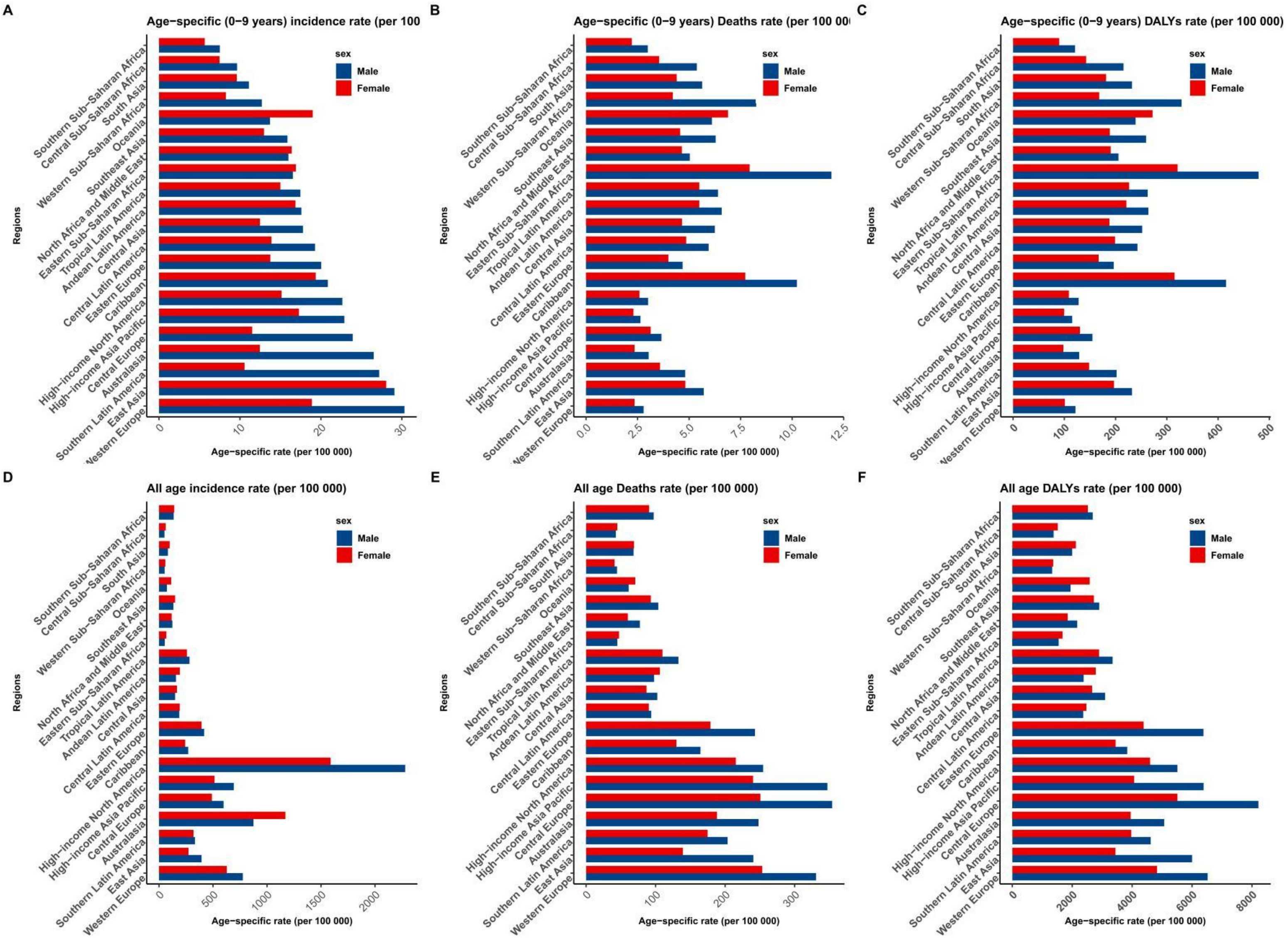

Supplementary Figure 3 Regional rates (per 100,00 population) of total cancer in 2019 among all ages and children aged 0-9 years

A. Incidence rates in aged 0-9 years in 2019; B. Deaths rates in aged 0-9 years in 2019; C. DALYs rates in aged 0-9 years in 2019; D. Incidence rates in all ages in 2019; E. Deaths rates in all ages in 2019; F. DAYLs rates in all ages in 2019.

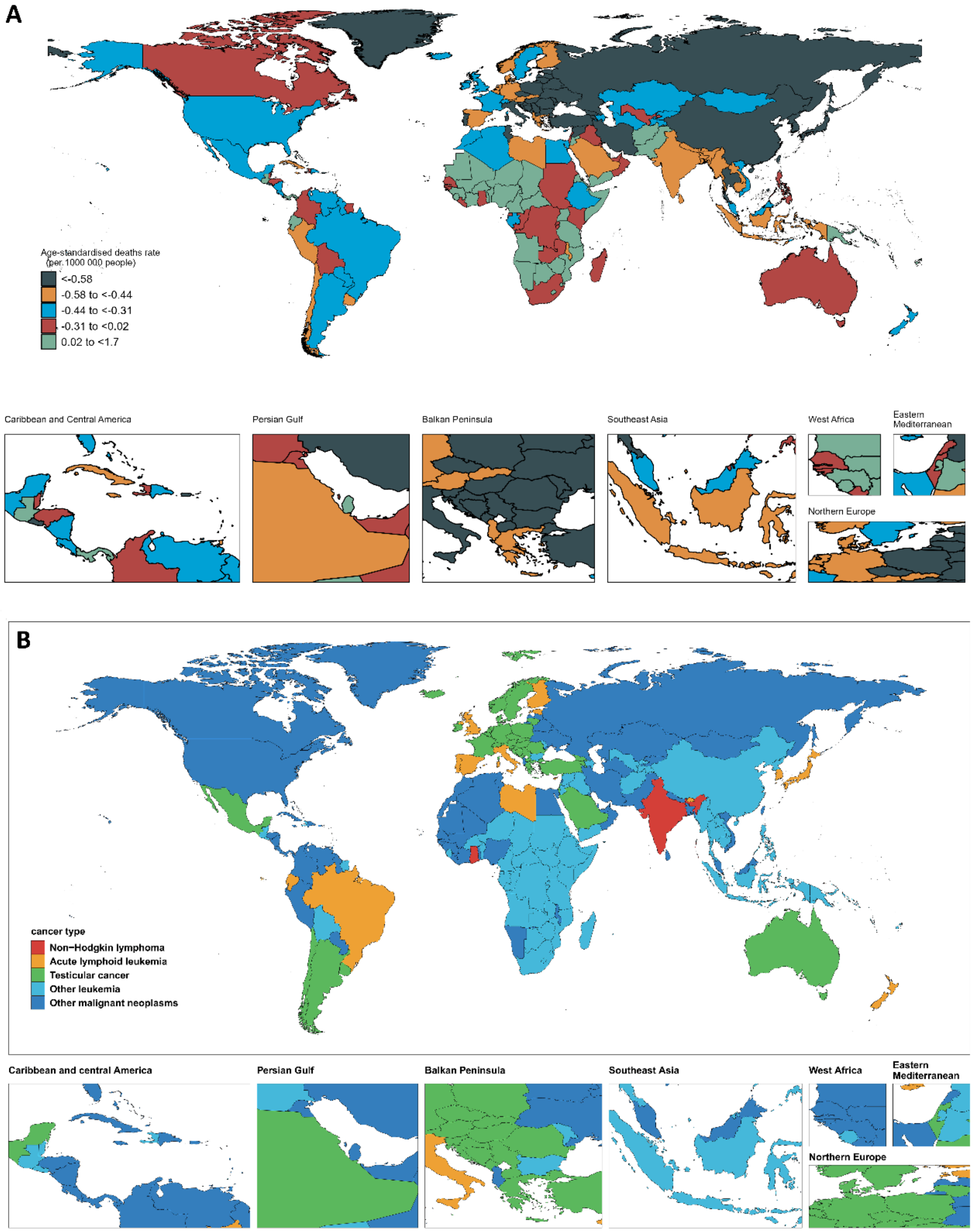

Supplementary Figure 4 Geographical distribution of childhood cancer for both sexes combined in 2019

Relative Changes in childhood cancer DALYs in Both Sexes for childhood Cancers in 195 Countries or Territories From 1990 to 2019. (B) The distribution of the highest kind of childhood cancer for both sexs in 195 countries or territories in 2019.

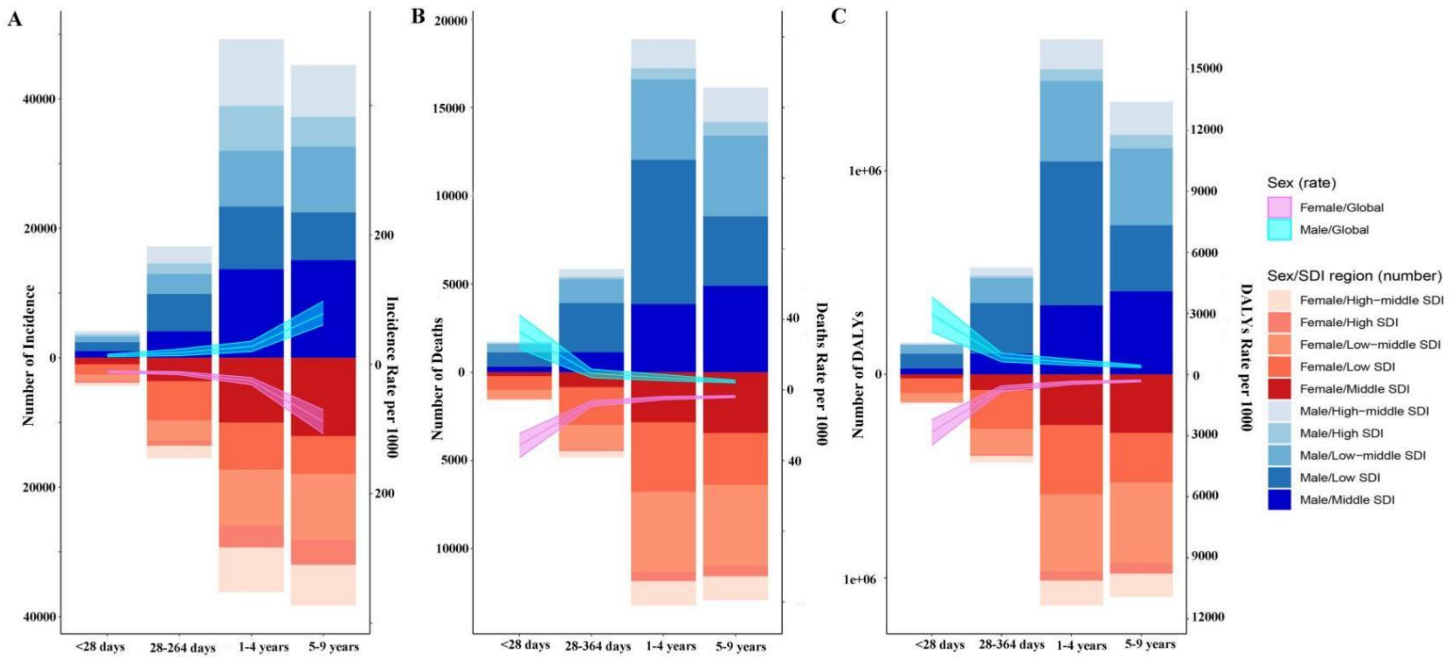

Supplementary Figure 51 The age distribution of childhood cancers incidence (A), deaths (B), and DALYs (C)

The bar was the number of childhood cancers of the incidence, deaths, and DALYs.

The line with 95% UI represented incidence rates, deaths rates, and DALYs rates for the childhood cancers. DALY = disability-adjusted life-years. SDI = sociodemographic index. GBD = Global Burden of Disease, Injuries, and Risk Factor Study.

Inter-category(colomn) ranking

	Brain and central nervous system cancer	Other malignant neoplasms	Acute lymphoid leukemia	Other leukemia	Acute myeloid leukemia	Non-Hodgkin lymphoma	Kidney cancer	Liver cancer	Chronic myeloid leukemia	Hodgkin lymphoma	Testicular cancer	Malignant skin melanoma	Colon and rectum cancer	Ovarian cancer	Nasopharynx cancer	Lip and oral cavity cancer	Thyroid cancer
**Global**																	
**SDI quintiles**																	
**High SDI**	5	5	5	5	5	5	5	5	5	5	5	5	5	5	5	5	5
**High-middle SDI**	4	4	4	4	4	4	4	4	4	4	4	4	4	4	4	4	4
**Middle SDI**	2	3	2	2	3	3	2	3	3	3	1	2	2	2	3	3	1
**Low-middle SDI**	3	1	1	1	1	1	1	1	1	2	2	1	3	3	2	2	3
**Low SDI**	1	2	3	3	2	2	3	2	2	1	3	3	1	1	1	1	2
**GBD super-region**																	
**Central Europe, Eastern Europe, and Central Asia**	7	7	7	7	7	7	7	7	7	6	7	6	7	7	5	7	7
**High-income**	6	6	6	6	6	6	6	6	6	7	6	2	5	6	7	4	6
**Latin America and Caribbean**	5	5	4	5	4	4	3	5	5	5	4	5	6	5	6	6	5
**North Africa and Middle East**	4	4	5	4	5	5	5	4	3	3	5	7	4	4	4	5	4
**South Asia**	1	2	3	3	2	2	4	3	2	1	2	3	1	1	1	1	1
**Southeast Asia, East Asia, and Oceania**	2	3	2	1	3	3	2	2	4	4	3	4	2	3	3	3	2
**Sub-Saharan Africa**	3	1	1	2	1	1	1	1	1	2	1	1	3	2	2	2	3
**Forty-seven most popubos countries**																	
**India**	1	2	2	3	1	1	3	1	2	3	1	1	1	1	1	2	2
**China**	2	4	1	1	2	2	1	2	10	5	3	2	2	3	3	3	1
**Pakistan**	3	1	4	6	3	4	6	3	3	1	2	5	3	2	2	1	3
**Nigeria**	4	3	8	9	4	3	2	4	5	2	5	4	4	5	5	5	5
**Indonesia**	7	9	7	2	6	6	5	5	15	15	8	12	5	4	7	6	6
**Ethiopia**	10	6	3	5	5	32	13	8	1	4	10	9	17	15	8	10	7
**Bangladesh**	6	7	11	26	12	13	26	9	9	6	14	14	8	7	4	4	11
**United Republic of Tanzania**	9	5	9	4	9	5	8	6	4	7	12	8	16	12	11	11	12
**Brazil**	5	10	6	16	7	8	4	18	22	12	9	6	10	8	14	9	10
**Philippines**	11	11	10	7	8	9	7	19	18	28	11	16	6	6	12	8	9
**Mexico**	15	17	5	23	10	15	9	15	28	11	4	10	14	10	22	13	13
**Uganda**	21	8	27	12	27	12	14	10	7	8	19	11	21	13	6	12	18
**Egypt**	16	15	20	22	20	17	16	7	17	30	13	24	7	9	24	16	4
**USA**	8	13	18	24	11	24	12	14	24	24	7	3	9	11	15	7	8
**Afghanistan**	12	14	12	10	15	19	25	22	8	9	27	28	24	31	20	18	19
**Iran**	17	25	21	25	17	25	21	26	16	17	16	25	11	18	27	17	16
**Mozambique**	19	12	13	8	18	34	19	13	6	10	29	18	43	28	29	29	20
**Turkey**	20	29	22	31	21	21	22	32	31	34	6	26	13	19	21	30	26
**Russian**	13	19	16	38	25	20	15	17	30	22	15	7	20	17	18	14	14
**Sudan**	14	23	17	13	16	22	17	31	11	13	26	32	23	29	26	23	22
**Myanmar**	23	24	19	11	24	7	18	11	23	36	36	37	15	14	25	22	23
**Viet Nam**	34	22	14	37	32	35	31	23	33	23	23	33	22	25	9	19	17
**Kenya**	26	27	25	20	29	18	38	28	14	21	41	36	32	33	16	20	29
**Iraq**	18	33	35	14	36	31	30	33	26	33	30	39	29	22	32	35	24
**Mali**	36	21	34	34	23	36	11	16	20	18	22	17	26	30	40	32	28
**Colombia**	27	32	15	33	26	23	23	24	34	27	25	20	30	26	38	38	31
**Madagascar**	41	20	30	21	37	30	34	25	12	16	44	35	42	39	28	41	38
**Yemen**	24	30	24	17	30	38	32	37	13	20	42	42	37	40	30	36	33
**Burkina Faso**	25	16	26	18	14	14	10	12	19	31	24	21	28	27	44	34	43
**Cameroon**	33	28	33	27	19	11	27	46	25	26	28	34	19	16	13	25	30
**Ghana**	30	31	37	42	22	10	20	20	38	40	33	38	12	20	36	37	15
**Argentina**	31	38	28	36	33	37	33	40	40	37	17	19	27	36	42	43	39
**Peru**	32	40	23	32	34	33	37	27	41	38	18	40	33	24	45	45	35
**Thailand**	40	41	44	19	42	40	42	42	42	45	38	43	35	35	37	42	41
**Morocco**	44	44	47	43	45	39	44	45	37	29	43	44	40	23	17	24	27
**Uzbekistan**	22	34	29	29	28	26	29	29	32	14	40	31	36	45	19	15	42
**Niger**	28	18	32	15	13	27	24	47	21	25	32	29	31	38	41	31	40
**Algeria**	42	37	45	39	31	16	40	34	27	19	34	41	34	34	10	39	32
**Japan**	37	35	36	40	35	42	39	30	35	39	35	30	25	37	34	21	25
**United Kingdom**	38	39	42	47	38	45	35	35	43	44	37	15	39	42	35	27	36
**Nepal**	39	26	38	44	40	29	47	21	29	32	45	45	45	32	31	26	45
**France**	29	43	39	35	39	41	36	38	44	42	31	13	41	43	39	40	34
**Malaysia**	47	47	31	41	43	43	46	44	46	46	46	46	44	44	23	46	46
**Germany**	35	42	43	45	41	44	43	39	39	41	20	23	38	41	43	44	37
**South Africa**	43	36	41	30	46	28	28	36	36	35	21	27	18	21	33	28	21
**Italy**	45	45	40	46	44	47	41	43	45	47	39	22	46	46	46	33	44
**Democratic People's Republic of Korea**	46	46	46	28	47	46	45	41	47	43	47	47	47	47	47	47	47

Intra-category(row) ranking

	Brain and central nervous system cancer	Other malignant neoplasms	Acute lymphoid leukemia	Other leukemia	Acute myeloid leukemia	Non-Hodgkin lymphoma	Kidney cancer	Liver cancer	Chronic myeloid leukemia	Hodgkin lymphoma	Testicular cancer	Malignant skin melanoma	Colon and rectum cancer	Ovarian cancer	Nasopharynx cancer	Lip and oral cavity cancer	Thyroid cancer
**Global**	2	1	3	4	5	6	7	8	9	10	11	12	13	14	15	16	17
**SDI quintiles**																	
**High SDI**	1	2	3	5	4	7	6	8	11	13	9	10	12	14	17	16	15
**High-middle SDI**	1	2	3	4	5	6	7	8	11	10	9	12	13	14	15	17	16
**Middle SDI**	1	2	3	4	5	6	7	8	10	11	9	14	12	13	15	17	16
**Low-middle SDI**	2	1	3	4	5	6	8	7	9	10	11	16	13	12	15	14	17
**Low SDI**	2	1	3	4	5	6	8	9	7	10	11	12	14	13	15	16	17
**GBD super-region**																	
**Central Europe, Eastern Europe, and Central Asia**	1	2	3	5	4	6	7	8	12	10	9	11	14	15	13	16	17
**High-income**	1	2	3	5	4	7	6	8	11	12	9	10	13	14	17	16	15
**Latin America and Caribbean**	2	3	1	5	4	6	7	8	11	10	9	12	14	13	16	17	15
**North Africa and Middle East**	1	2	3	4	5	6	7	8	9	11	10	15	12	13	16	17	14
**South Asia**	2	1	3	6	4	5	10	7	8	9	11	16	14	12	15	13	17
**Southeast Asia, East Asia, and Oceania**	1	3	4	2	5	6	8	7	10	11	9	14	12	13	15	17	16
**Sub-Saharan Africa**	3	1	2	4	5	6	7	9	8	10	11	12	13	14	15	17	16
**Forty-seven most popubos countries**																	
**India**	1	2	3	6	4	5	9	7	8	10	11	16	12	13	15	14	17
**China**	1	4	3	2	5	6	7	8	11	10	9	13	12	15	14	17	16
**Pakistan**	2	1	3	5	4	7	10	9	8	6	11	16	15	13	14	12	17
**Nigeria**	2	1	5	7	4	3	6	9	10	8	11	12	13	14	15	16	17
**Indonesia**	4	1	3	2	5	6	7	8	10	13	9	14	11	12	15	17	16
**Ethiopia**	5	2	1	4	6	10	8	7	3	9	11	12	16	15	13	17	14
**Bangladesh**	2	1	3	7	4	6	9	5	8	10	11	16	15	12	13	14	17
**United Republic of Tanzania**	3	1	4	2	6	5	9	8	7	10	11	12	15	13	14	16	17
**Brazil**	1	2	3	7	4	6	5	8	12	11	9	10	14	13	16	17	15
**Philippines**	4	1	3	2	5	6	7	8	9	13	10	14	11	12	15	17	16
**Mexico**	3	2	1	6	4	5	7	8	12	10	9	11	14	13	17	16	15
**Uganda**	3	1	4	2	7	5	9	6	8	10	11	12	15	14	13	16	17
**Egypt**	2	1	3	6	5	7	8	4	9	14	10	15	12	13	17	16	11
**USA**	1	2	3	5	4	8	6	7	11	13	9	10	12	14	16	17	15
**Afghanistan**	2	1	4	3	5	6	8	10	7	9	11	12	13	14	15	17	16
**Iran**	1	2	3	5	4	6	7	9	8	11	10	14	12	13	17	16	15
**Mozambique**	3	1	4	2	5	9	8	7	6	10	11	12	16	13	15	17	14
**Turkey**	1	2	3	5	4	6	7	9	10	11	8	14	12	13	15	17	16
**Russian**	1	2	3	8	4	6	5	7	12	11	9	10	14	13	15	17	16
**Sudan**	1	2	3	4	5	6	7	9	8	10	11	13	12	14	15	17	16
**Myanmar**	5	2	4	1	7	3	8	6	9	13	10	14	11	12	15	16	17
**Viet Nam**	3	1	2	6	4	5	7	8	12	10	9	15	13	14	11	17	16
**Kenya**	2	1	4	3	6	5	9	8	7	10	11	13	14	15	12	16	17
**Iraq**	1	3	4	2	5	6	7	8	9	11	10	14	13	12	16	17	15
**Mali**	2	1	5	7	3	8	4	6	9	10	11	12	13	14	17	16	15
**Colombia**	3	2	1	7	4	5	6	8	12	10	9	11	14	13	16	17	15
**Madagascar**	4	1	3	2	5	6	8	9	7	10	11	12	15	13	14	17	16
**Yemen**	1	2	4	3	5	6	8	9	7	10	11	13	12	14	15	17	16
**Burkina Faso**	2	1	4	3	5	6	7	8	9	11	10	12	13	14	17	15	16
**Cameroon**	3	1	6	5	4	2	7	14	8	10	9	15	13	11	12	16	17
**Ghana**	3	1	5	8	4	2	6	7	12	14	9	13	10	11	16	17	15
**Argentina**	1	3	2	5	4	6	7	9	12	11	8	10	13	14	16	17	15
**Peru**	2	3	1	4	5	6	8	7	12	11	9	14	13	10	16	17	15
**Thailand**	2	3	4	1	5	6	7	8	10	13	9	14	12	11	15	17	16
**Morocco**	2	1	4	6	5	3	7	9	12	8	10	17	14	11	13	15	16
**Uzbekistan**	1	2	3	5	4	6	7	8	10	9	11	12	15	16	13	14	17
**Niger**	3	1	5	2	4	7	6	11	8	10	9	12	13	14	17	15	16
**Algeria**	2	1	5	6	4	3	7	8	10	9	11	15	14	13	12	17	16
**Japan**	2	1	3	5	4	7	8	6	10	13	9	11	12	14	17	15	16
**United Kingdom**	1	2	3	8	4	6	5	7	11	13	10	9	12	14	16	15	17
**Nepal**	2	1	3	7	6	4	9	5	8	10	11	16	15	12	13	14	17
**France**	1	2	3	4	5	6	7	8	11	12	9	10	13	14	16	17	15
**Malaysia**	3	2	1	5	4	6	7	8	12	11	9	15	13	14	10	17	16
**Germany**	1	2	3	7	4	5	6	9	11	13	8	10	12	14	16	17	15
**South Africa**	2	1	4	3	7	5	6	8	14	10	9	13	11	12	17	16	15
**Italy**	2	1	3	7	4	6	5	8	11	12	9	10	13	15	17	14	16
**Democratic People's Republic of Korea**	2	3	4	1	5	6	7	8	10	9	11	12	14	13	15	17	16

Supplementary Figure 6 Childhood cancers ranked by number of DALYs for both sex combined, 2019

Inter-category ranking refer to ranking vertically (ranking between the SDI quintiles, between the GBD super-regions, and between countries). Intra-category ranking refer to ranking horizontally (ranking within each SDI quintile, within each GBD super-region, and within each country)

B Male

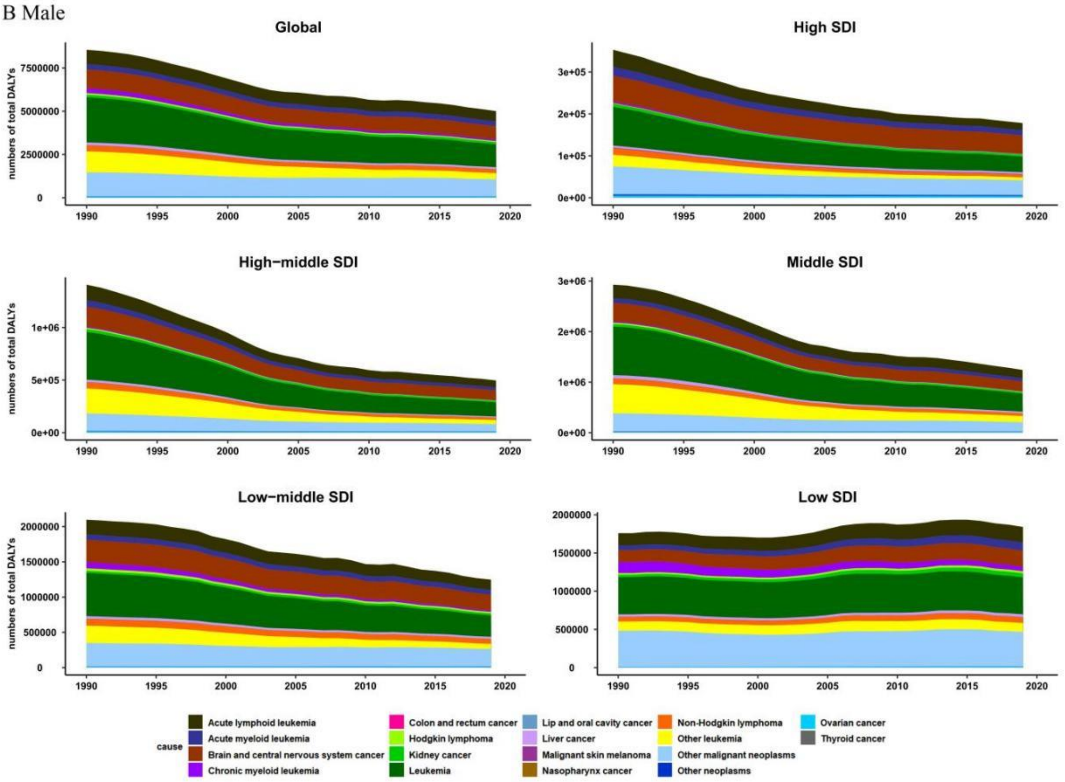

Supplementary Figure 7 Numbers of total DALYs by different cancer types in children aged 0–9 years from 1990 to 2019 by sex and GBD SDI regions

(A) Females (B) Males. GBD = Global Burden of Diseases, Injuries, and Risk Factors Study

B Male

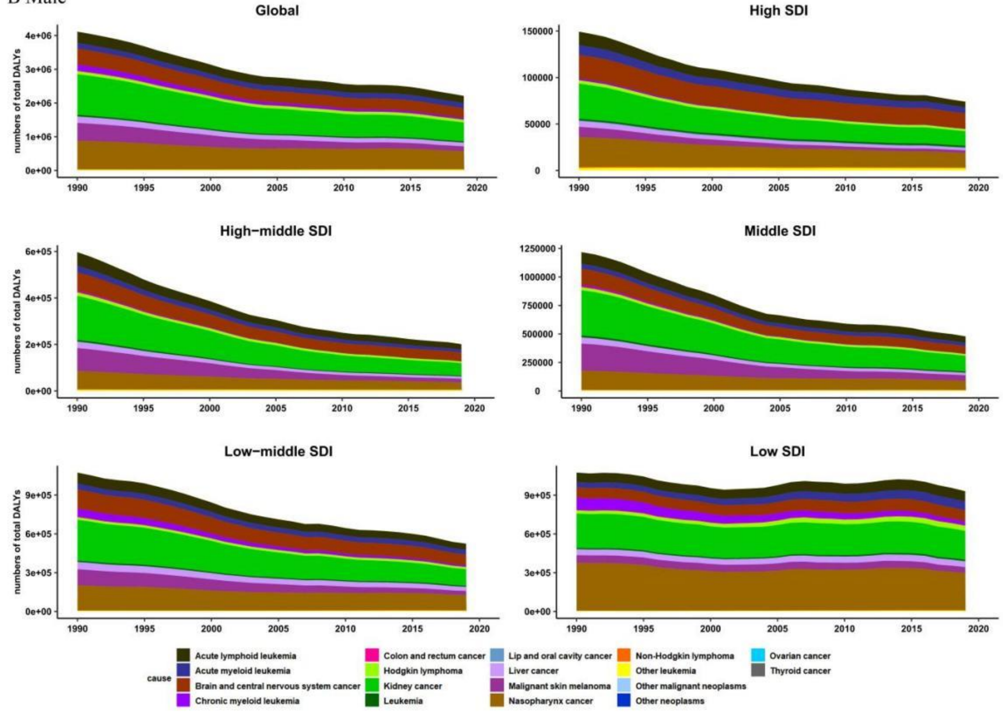

Supplementary Figure 8 Numbers of total DALYs by different cancer types in children aged 1-4 years from 1990 to 2019 by sex and GBD SDI regions

(A) Females (B) Males. GBD = Global Burden of Diseases, Injuries, and Risk Factors Study

B Male

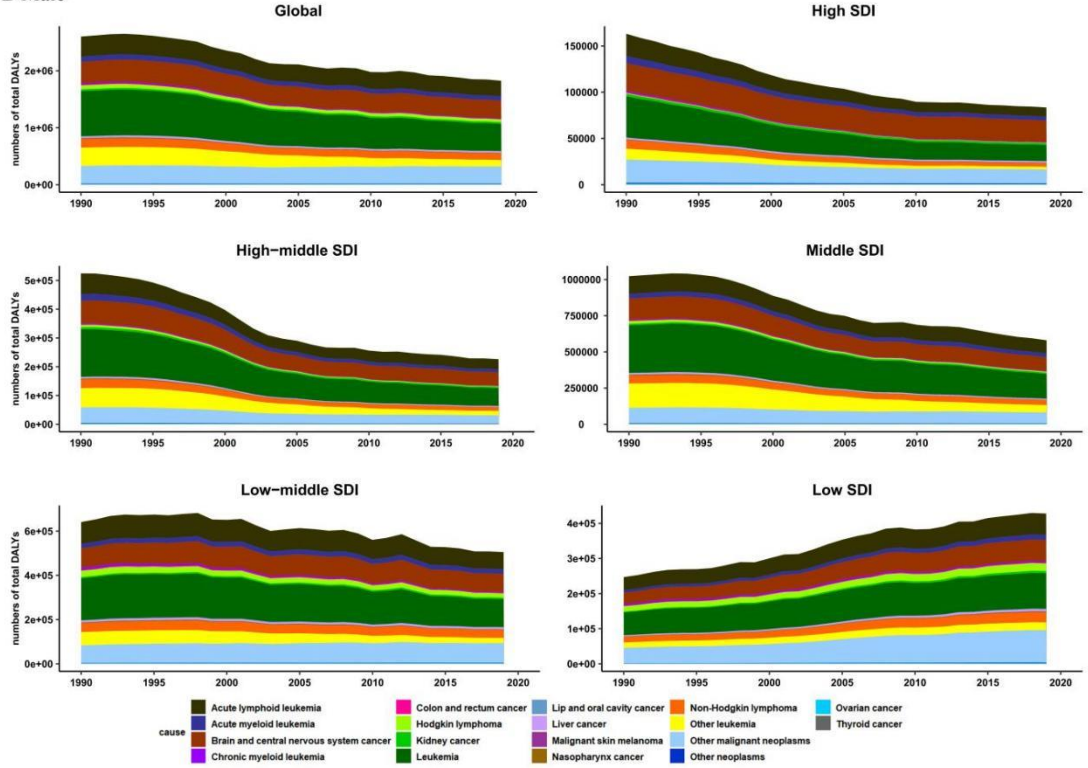

Supplementary Figure 9 Numbers of total DALYs by different cancer types in children aged 5-9 years from 1990 to 2019 by sex and GBD SDI regions

(A) Females (B) Males. GBD = Global Burden of Diseases, Injuries, and Risk Factors Study

B Male

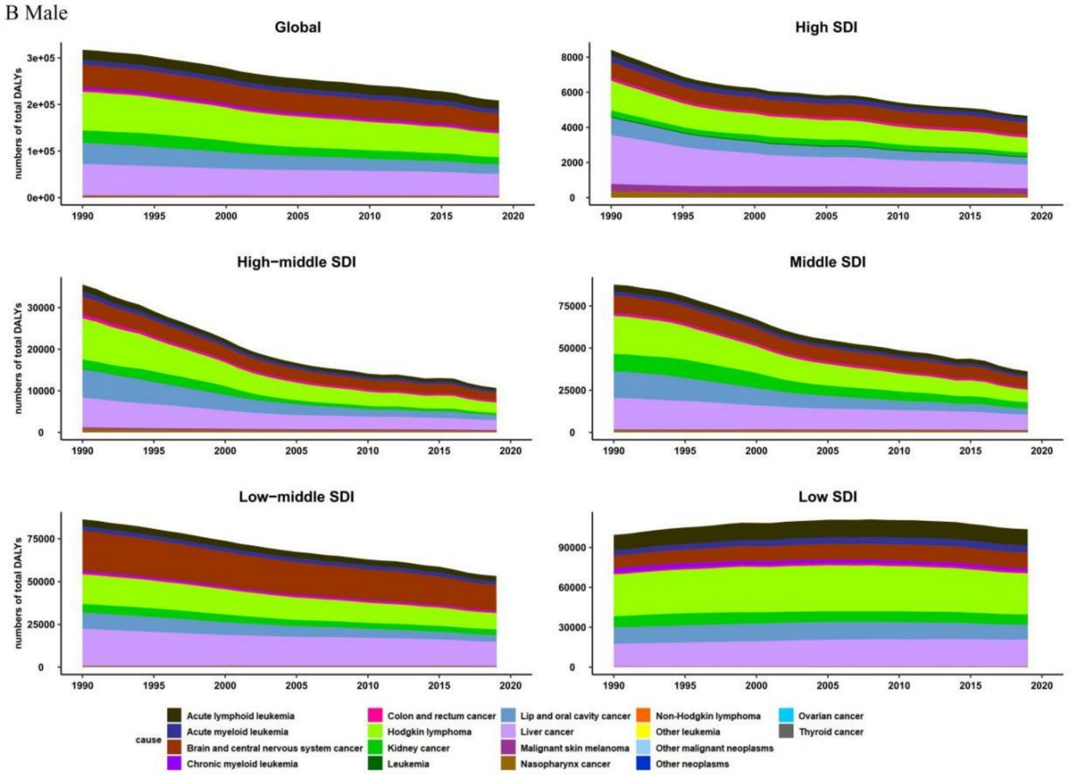

Supplementary Figure 10 Numbers of total DALYs by different cancer types in children aged < 28 days from 1990 to 2019 by sex and GBD SDI regions

(A) Females (B) Males. GBD = Global Burden of Diseases, Injuries, and Risk Factors Study

B Male

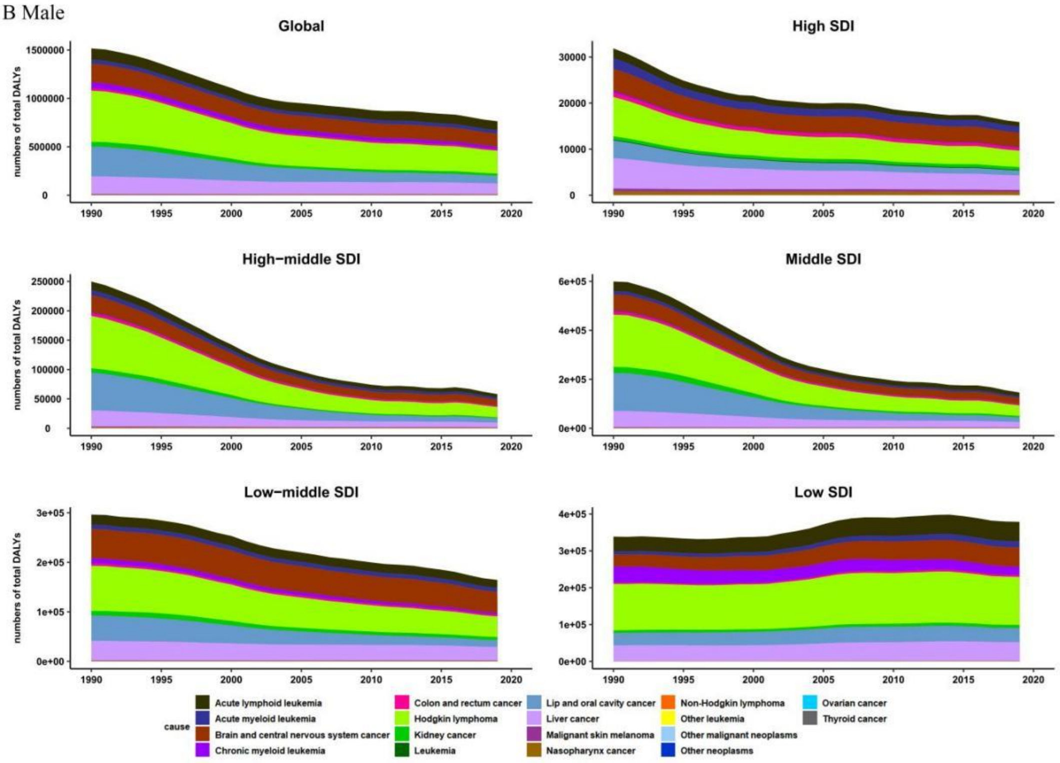

Supplementary Figure 11 Numbers of total DALYs by different cancer types in children aged 28-365 days from 1990 to 2019 by sex and GBD SDI regions

(A) Females (B) Males. GBD = Global Burden of Diseases, Injuries, and Risk Factors Study

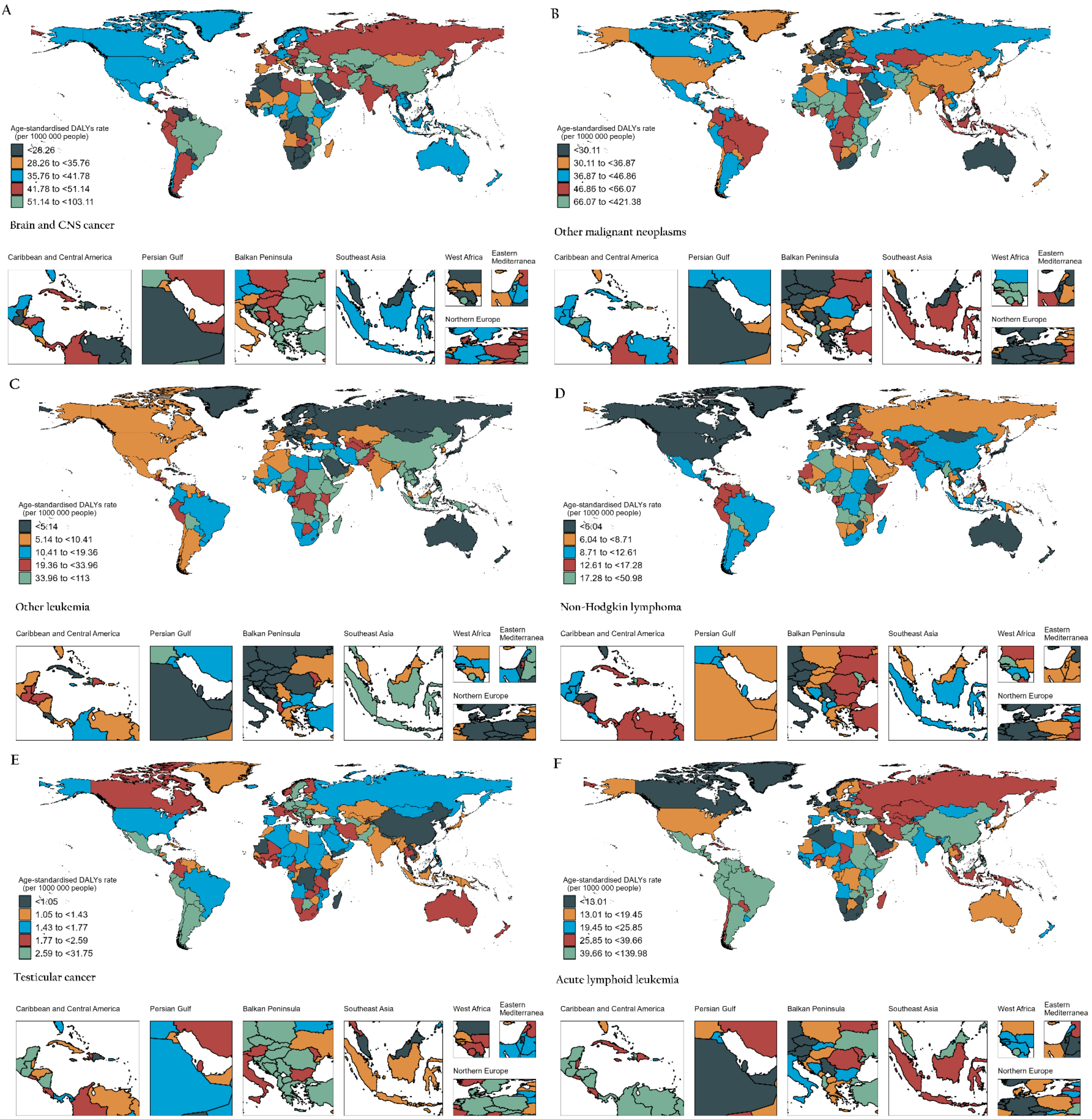

Supplementary Figure 12 Geographical age-standardized DALYs rates distribution of specific cancer among children for both sexes combined in 2019.

(A) Brain and CNS cancer; (B) other malignant neoplasm; (C) other leukemia; (D) Non-Hodgkin leukemia; (E) testicular cancer; (F) acute lymphoid leukemia.

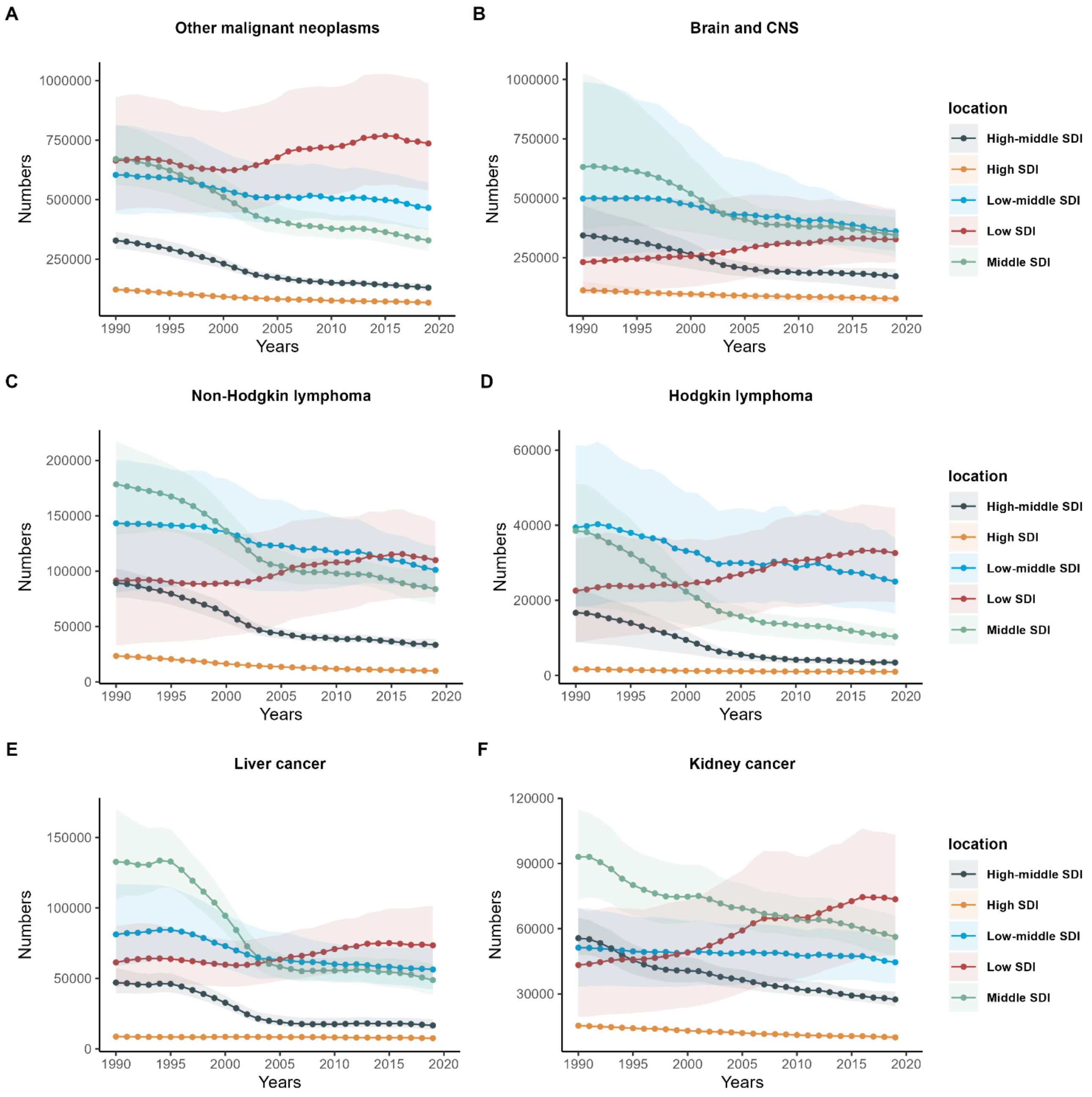

Supplementary Figure 13 Trend in specific cancer among children aged 0-9 years in DALYs numbers in both sexes from 1990 to 2019.

(A) other malignant neoplasm; (B) brain and CNS cancer; (C) Non-Hodgkin leukemia; (D) Hodgkin leukemia; (E) liver cancer; (F) kidney cancer.

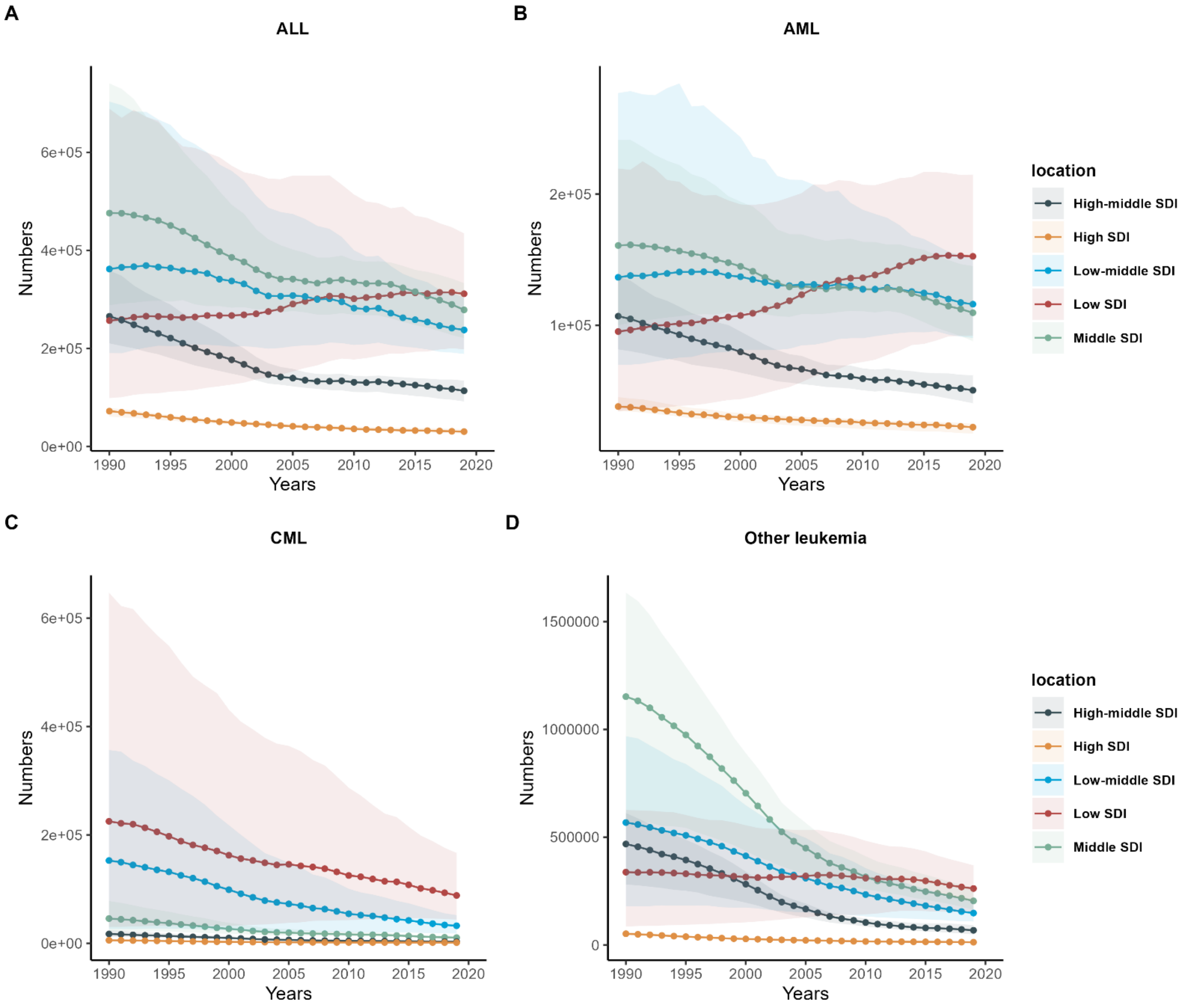

Supplementary Figure 14 Trend in specific cancer among children aged 0-9 years in DALYs numbers in both sexes from 1990 to 2019.

(A) acute lymphoid leukemia (ALL); (B) acute myeloid leukemia (AML); (C) chronic myeloid leukemia (CML); (D) other leukemia.

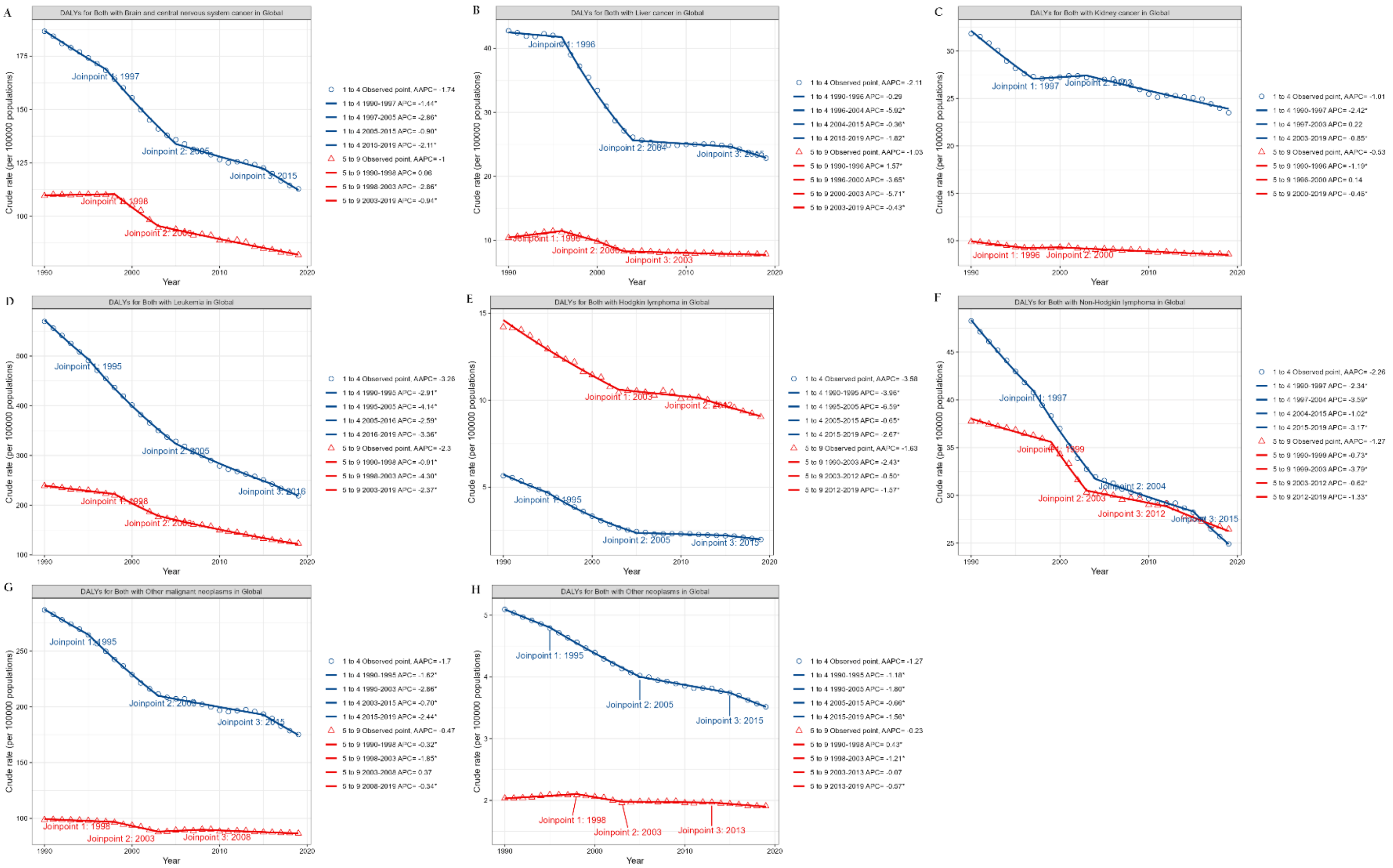

Supplementary Figure 15 Joinpoint regression analysis of global specific cancers DALYs rates in adolescents and young adults aged 1 to 4 and 5 to 9 years from 1990 to 2019.

(A) Brain and CNS cancer; (B) liver cancer; (C) kidney cancer; (D) leukemia; (E) Hodgkin leukemia; (F) Non-Hodgkin leukemia; (G) other malignant neoplasm; (H) other neoplasms.

Supplementary Table 1 Worldwide Incidence, Deaths, and DALYs and rates by SDI and regions for total cancers in 2019 in children younger than 10 years and the change in the trends from 1990 to 2019 in Males

Location	Incidence			Deaths			DALYs		
	Number	Rate per 10,000	Change in rates(%)	Number	Rate per 10,000	Change in rates(%)	Number	Rate per 10,000	Change in rates(%)
Global	115863 (93545-140358)	17.03 (13.75-20.63)	-0.28 (-0.5-0.05)	42600 (35630-50855)	6.26 (5.24-7.47)	-0.43 (-0.58--0.14)	3669520 (3072732-4387143)	256.01 (214.37-306.08)	-0.42 (-0.57--0.12)
**Social-demograghic index**									
High SDI	13446 (7672-20902)	24.49 (13.98-38.08)	-0.03 (-0.24-0.24)	1557 (1336-1715)	2.84 (2.43-3.12)	-0.43 (-0.54--0.37)	138485 (116256-155587)	121.52 (102.01-136.53)	-0.41 (-0.53--0.34)
High-middle SDI	21441 (15814-27530)	24.97 (18.42-32.06)	-0.16 (-0.4-0.13)	4118 (3432-4804)	4.8 (4-5.6)	-0.54 (-0.65--0.36)	358509 (297748-418569)	198.69 (165.02-231.98)	-0.53 (-0.64--0.35)
Middle SDI	33894 (27835-40316)	17.6 (14.45-20.93)	-0.35 (-0.58-0.01)	10209 (8525-11955)	5.3 (4.43-6.21)	-0.52 (-0.66--0.24)	877552 (732225-1028598)	217.7 (181.64-255.17)	-0.51 (-0.65--0.23)
Low-middle SDI	22757 (18473-28206)	12.72 (10.33-15.77)	-0.3 (-0.55-0.19)	10978 (8882-13547)	6.14 (4.97-7.57)	-0.41 (-0.6-0)	939119 (759629-1156436)	250.72 (202.8-308.73)	-0.4 (-0.59-0.03)
Low SDI	24249 (17706-31925)	14.46 (10.56-19.04)	-0.29 (-0.55-0.35)	15705 (11394-20835)	9.37 (6.8-12.43)	-0.43 (-0.61--0.01)	1352982 (980942-1798495)	375.45 (272.21-499.08)	-0.41 (-0.6-0.02)
**Region**									
**Africa**									
Central Sub-Saharan Africa	1946 (1209-3217)	9.64 (5.99-15.94)	-0.49 (-0.71-0.15)	1085 (672-1676)	5.38 (3.33-8.31)	-0.55 (-0.72--0.11)	93102 (57441-143997)	215.36 (132.87-333.08)	-0.53 (-0.71--0.06)
Eastern Sub-Saharan Africa	10301 (6762-14457)	16.52 (10.85-23.19)	-0.38 (-0.68-0.42)	7422 (5340-9857)	11.9 (8.56-15.81)	-0.55 (-0.73--0.15)	642737 (462375-855512)	478.79 (344.43-637.29)	-0.53 (-0.72--0.12)
North Africa and Middle East	9795 (7586-12379)	15.98 (12.38-20.19)	-0.19 (-0.49-0.37)	3087 (2328-3855)	5.04 (3.8-6.29)	-0.43 (-0.63--0.04)	264738 (198936-330460)	205.59 (154.49-256.62)	-0.42 (-0.62--0.01)
Southern Sub-Saharan Africa	608 (475-789)	7.5 (5.86-9.74)	-0.12 (-0.38-0.25)	243 (189-307)	2.99 (2.34-3.79)	-0.27 (-0.48-0.07)	20651 (16089-26195)	120.92 (94.21-153.38)	-0.26 (-0.48-0.08)

Western Sub-Saharan Africa	8906 (5700-13104)	12.69 (8.12-18.67)	-0.09 (-0.42-0.39)	5792 (3682-8551)	8.25 (5.25-12.18)	-0.24 (-0.48-0.1)	498893 (316511-738109)	328.82 (208.61-486.49)	-0.22 (-0.46-0.14)
**America**									
Andean Latin America	1114 (787-1541)	17.58 (12.42-24.32)	-0.2 (-0.65-0.39)	417 (292-563)	6.58 (4.61-8.88)	-0.41 (-0.68--0.07)	35522 (24789-47864)	263.8 (184.09-355.46)	-0.4 (-0.68--0.05)
Central Latin America	4267 (2637-6873)	19.27 (11.91-31.05)	-0.07 (-0.4-0.4)	1316 (1040-1679)	5.94 (4.7-7.58)	-0.31 (-0.47--0.09)	112723 (88825-143224)	242.61 (191.17-308.25)	-0.3 (-0.46--0.06)
High-income North America	4952 (2631-8117)	22.64 (12.03-37.11)	-0.11 (-0.33-0.17)	659 (579-724)	3.01 (2.65-3.31)	-0.33 (-0.43--0.26)	58146 (50545-64540)	127.89 (111.17-141.95)	-0.31 (-0.41--0.23)
Southern Latin America	1382 (540-3293)	27.2 (10.64-64.83)	0.23 (-0.35-1.48)	244 (209-289)	4.81 (4.12-5.69)	-0.39 (-0.49--0.27)	21215 (17935-25300)	201.84 (170.64-240.71)	-0.36 (-0.48--0.22)
Tropical Latin America	2915 (2015-4013)	17.46 (12.07-24.03)	-0.15 (-0.41-0.22)	1069 (838-1313)	6.4 (5.02-7.86)	-0.34 (-0.54--0.11)	91345 (71742-112219)	262.48 (206.15-322.46)	-0.34 (-0.54--0.11)
Caribbean	834 (539-1237)	20.84 (13.46-30.89)	-0.07 (-0.3-0.22)	409 (243-657)	10.23 (6.07-16.41)	-0.16 (-0.37-0.09)	35015 (20773-56060)	415.51 (246.5-665.23)	-0.14 (-0.35-0.12)
**Asia**									
Central Asia	1737 (1217-2451)	17.77 (12.44-25.07)	-0.34 (-0.51--0.1)	611 (505-750)	6.24 (5.16-7.67)	-0.37 (-0.51--0.2)	52014 (42850-64026)	251.77 (207.41-309.92)	-0.35 (-0.5--0.17)
East Asia	24957 (19332-32640)	29.07 (22.52-38.02)	-0.32 (-0.59-0.35)	4903 (3724-6451)	5.71 (4.34-7.51)	-0.63 (-0.76--0.27)	428514 (323807-566018)	232 (175.31-306.44)	-0.63 (-0.76--0.25)
High-income Asia Pacific	1790 (1230-2520)	22.9 (15.74-32.24)	-0.07 (-0.34-0.29)	206 (174-231)	2.64 (2.22-2.96)	-0.52 (-0.66--0.41)	18409 (15453-20628)	115.1 (96.62-128.97)	-0.51 (-0.65--0.41)
South Asia	19470 (15306-24626)	11.09 (8.72-14.03)	-0.17 (-0.47-0.5)	9891 (7854-12329)	5.63 (4.47-7.02)	-0.35 (-0.58-0.17)	843552 (669367-1052495)	231.8 (183.93-289.21)	-0.33 (-0.57-0.21)
Southeast Asia	9080 (7389-11848)	15.86 (12.9-20.69)	-0.27 (-0.56-0.27)	3603 (2921-4668)	6.29 (5.1-8.15)	-0.38 (-0.59-0.05)	308307 (250276-397882)	259.34 (210.53-334.69)	-0.37 (-0.59-0.07)

**Europe**									
Central Europe	1424 (680-2669)	23.92 (11.42-44.82)	-0.06 (-0.45-0.58)	218 (178-262)	3.66 (2.99-4.4)	-0.57 (-0.68--0.46)	19058 (15421-23256)	154.64 (125.13-188.71)	-0.57 (-0.68--0.45)
Eastern Europe	2613 (1574-3972)	20.04 (12.06-30.45)	-0.26 (-0.5-0.05)	611 (514-722)	4.68 (3.94-5.53)	-0.54 (-0.63--0.44)	52776 (44109-62487)	196.22 (164-232.33)	-0.53 (-0.62--0.43)
Western Europe	7029 (3645-11306)	30.31 (15.72-48.75)	-0.02 (-0.28-0.33)	648 (533-726)	2.79 (2.3-3.13)	-0.49 (-0.6--0.42)	58405 (47094-67539)	121.78 (98.2-140.83)	-0.47 (-0.59--0.39)
**Oceania**									
Oceania	244 (141-408)	13.7 (7.93-22.89)	0.01 (-0.3-0.46)	109 (65-179)	6.11 (3.66-10.02)	-0.04 (-0.32-0.37)	9326 (5555-15344)	238.9 (142.3-393.05)	-0.04 (-0.33-0.36)
Australasia	498 (199-1009)	26.51 (10.6-53.77)	0.24 (-0.3-1.11)	57 (46-66)	3.04 (2.47-3.54)	-0.41 (-0.54--0.3)	5071 (4041-6049)	128.98 (102.79-153.86)	-0.38 (-0.53--0.26)

SDI = sociodemographic index.

Supplementary Table 2 Worldwide incidence, Deaths, and DALYs and rates by SDI and regions for total cancers in 2019 in children younger than 10 years and the change in the trends from 1990 to 2019 in Females

Location	Incidence			Deaths			DALYs		
	**Number**	**Rate per 10,000**	**Change in rates(%)**	**Number**	**Rate per 10,000**	**Change in rates(%)**	**Number**	**Rate per 10,000**	**Change in rates(%)**
Global	90739 (79425-103836)	14.24 (12.46-16.3)	-0.42 (-0.57--0.17)	29590 (25638-34086)	4.64 (4.02-5.35)	-0.49 (-0.6--0.3)	2554489 (2211895-2948279)	190.37 (164.84-219.71)	-0.48 (-0.6--0.28)
**Social-demograghic index**									
High SDI	8473 (7207-9819)	16.26 (13.83-18.84)	-0.15 (-0.28--0.01)	1271 (1110-1367)	2.44 (2.13-2.62)	-0.43 (-0.52--0.37)	111775 (97690-121219)	103.32 (90.3-112.05)	-0.41 (-0.51--0.35)
High-middle SDI	15615 (13508-17743)	19.87 (17.19-22.57)	-0.33 (-0.5--0.11)	3168 (2800-3508)	4.03 (3.56-4.46)	-0.58 (-0.66--0.48)	276204 (243304-307079)	167.33 (147.4-186.04)	-0.57 (-0.65--0.47)
Middle SDI	26768 (23446-30000)	15.15 (13.27-16.98)	-0.47 (-0.62--0.22)	7332 (6500-8288)	4.15 (3.68-4.69)	-0.58 (-0.67--0.44)	632511 (560286-713870)	170.89 (151.37-192.87)	-0.58 (-0.67--0.43)
Low-middle SDI	19054 (15405-23262)	11.34 (9.17-13.85)	-0.43 (-0.62--0.04)	7994 (6587-9597)	4.76 (3.92-5.71)	-0.47 (-0.62--0.16)	684965 (563804-821139)	195 (160.51-233.77)	-0.45 (-0.61--0.13)
Low SDI	20757 (15692-26981)	12.86 (9.72-16.72)	-0.39 (-0.62-0.25)	9798 (7743-12204)	6.07 (4.8-7.56)	-0.41 (-0.6-0.13)	846808 (668230-1056615)	244.49 (192.93-305.06)	-0.4 (-0.59-0.17)
**Region**									
**Africa**									
Central Sub-Saharan Africa	1488 (980-2297)	7.47 (4.92-11.53)	-0.55 (-0.74-0.13)	706 (482-990)	3.54 (2.42-4.97)	-0.54 (-0.72-0.01)	60643 (41222-84978)	142.56 (96.9-199.76)	-0.52 (-0.7-0.05)
Eastern Sub-Saharan Africa	10246 (6767-14024)	16.91 (11.17-23.14)	-0.46 (-0.7-0.3)	4808 (3653-6161)	7.93 (6.03-10.17)	-0.46 (-0.67-0.18)	418596 (317590-537287)	320.85 (243.43-411.82)	-0.45 (-0.66-0.22)
North Africa and Middle East	9494 (7751-11423)	16.38 (13.38-19.71)	-0.35 (-0.56-0.11)	2692 (2157-3216)	4.65 (3.72-5.55)	-0.46 (-0.63--0.12)	232090 (186206-277715)	190.51 (152.85-227.96)	-0.45 (-0.62--0.09)

Southern Sub-Saharan Africa	451 (358-564)	5.63 (4.48-7.05)	-0.15 (-0.37-0.18)	178 (143-220)	2.22 (1.79-2.75)	-0.18 (-0.38-0.12)	15157 (12211-18726)	90.03 (72.53-111.23)	-0.17 (-0.38-0.13)
Western Sub-Saharan Africa	5678 (4069-7558)	8.26 (5.92-10.99)	-0.08 (-0.34-0.29)	2894 (2136-3816)	4.21 (3.11-5.55)	-0.17 (-0.4-0.16)	248720 (183103-328599)	167.82 (123.55-221.72)	-0.15 (-0.38-0.19)
**America**									
Andean Latin America	1020 (716-1387)	16.84 (11.83-22.9)	-0.26 (-0.55-0.18)	333 (243-431)	5.49 (4.01-7.12)	-0.38 (-0.64--0.08)	28479 (20771-36992)	221.04 (161.22-287.11)	-0.37 (-0.63--0.07)
Central Latin America	2945 (2368-3641)	13.88 (11.16-17.16)	-0.27 (-0.42--0.09)	1032 (826-1256)	4.86 (3.89-5.92)	-0.39 (-0.54--0.23)	88502 (70667-107982)	198.7 (158.65-242.43)	-0.37 (-0.53--0.21)
High-income North America	3164 (2646-3814)	15.13 (12.65-18.24)	-0.19 (-0.33--0.03)	541 (475-581)	2.59 (2.27-2.78)	-0.33 (-0.42--0.25)	47258 (41592-50884)	108.81 (95.77-117.16)	-0.31 (-0.4--0.23)
Southern Latin America	517 (429-616)	10.57 (8.77-12.6)	-0.28 (-0.41--0.12)	175 (156-199)	3.59 (3.19-4.07)	-0.44 (-0.52--0.36)	15008 (13285-17071)	148.25 (131.23-168.64)	-0.43 (-0.51--0.34)
Tropical Latin America	2404 (1933-2899)	14.99 (12.05-18.08)	-0.32 (-0.49--0.13)	881 (692-1059)	5.49 (4.31-6.6)	-0.42 (-0.58--0.25)	75510 (59236-90727)	226.18 (177.44-271.77)	-0.43 (-0.59--0.25)
Caribbean	749 (463-1121)	19.35 (11.95-28.95)	-0.32 (-0.54--0.05)	299 (189-447)	7.73 (4.89-11.54)	-0.31 (-0.51--0.05)	25638 (16129-38319)	314.92 (198.12-470.69)	-0.3 (-0.51--0.03)
**Asia**									
Central Asia	1136 (949-1391)	12.46 (10.41-15.26)	-0.48 (-0.58--0.35)	425 (359-502)	4.66 (3.94-5.51)	-0.41 (-0.52--0.27)	36192 (30678-42729)	188 (159.36-221.95)	-0.4 (-0.51--0.26)
East Asia	20629 (16833-24323)	28.05 (22.89-33.08)	-0.43 (-0.62--0.08)	3540 (3028-4152)	4.81 (4.12-5.65)	-0.68 (-0.77--0.52)	312189 (265647-365517)	196.91 (167.55-230.55)	-0.68 (-0.77--0.52)
High-income Asia Pacific	1282 (1101-1488)	17.27 (14.84-20.05)	-0.16 (-0.31-0.03)	171 (154-184)	2.3 (2.07-2.48)	-0.51 (-0.59--0.44)	15125 (13640-16389)	99.5 (89.73-107.81)	-0.5 (-0.59--0.44)

South Asia	15505 (11784-19594)	9.6 (7.3-12.13)	-0.31 (-0.55-0.26)	7107 (5692-8720)	4.4 (3.52-5.4)	-0.41 (-0.6-0.01)	606824 (485840-746836)	181.44 (145.27-223.31)	-0.39 (-0.58-0.05)
Southeast Asia	6998 (5564-9042)	12.96 (10.31-16.75)	-0.43 (-0.65-0)	2465 (2012-3045)	4.57 (3.73-5.64)	-0.45 (-0.61--0.09)	211467 (172133-261108)	188.66 (153.57-232.95)	-0.44 (-0.61--0.07)
**Europe**									
Central Europe	646 (550-756)	11.51 (9.79-13.47)	-0.3 (-0.42--0.16)	176 (152-204)	3.13 (2.71-3.64)	-0.53 (-0.64--0.43)	15174 (13132-17583)	130.44 (112.89-151.15)	-0.53 (-0.63--0.43)
Eastern Europe	1698 (1456-1952)	13.75 (11.78-15.8)	-0.37 (-0.46--0.27)	493 (425-560)	3.99 (3.44-4.53)	-0.51 (-0.59--0.42)	42471 (36680-48298)	166.85 (144.1-189.75)	-0.51 (-0.59--0.42)
Western Europe	4155 (3362-4933)	18.87 (15.27-22.41)	-0.14 (-0.34-0.05)	517 (425-568)	2.35 (1.93-2.58)	-0.49 (-0.61--0.41)	45925 (37706-50729)	100.87 (82.81-111.42)	-0.47 (-0.6--0.39)
**Oceania**									
Oceania	313 (184-518)	18.98 (11.15-31.42)	0.05 (-0.31-0.57)	114 (72-175)	6.89 (4.35-10.6)	-0.01 (-0.32-0.47)	9863 (6206-15247)	272.12 (171.23-420.67)	-0.01 (-0.32-0.47)
Australasia	222 (160-300)	12.45 (8.99-16.86)	-0.12 (-0.32-0.16)	42 (35-48)	2.35 (1.96-2.69)	-0.41 (-0.53--0.29)	3658 (3012-4185)	98.12 (80.78-112.26)	-0.4 (-0.52--0.28)

SDI = sociodemographic index.

Supplementary Table 3 Average Annual Percent Change of Epidemiological Metrics of Total cancers in age-standardized and in children younger than 10 years in Global from 1990 to 2019 (Supplementary Table)

Metric	Total cancers in all ages			Total cancers in children		
	Both	Male	Female	Both	Male	Female
**Prevalence rate**	0.64(0.57, 0.71)	0.83(0.74, 0.92)	0.45(0.39, 0.51)	-1.37(-1.49, -1.26)	-0.94(-1.06, -0.82)	-1.85(-2.00, -1.70)
**Incidence rate**	0.37(0.27, 0.46)	0.42(0.31,0.53)	0.26(0.17, 0.34)	-1.49(-1.60, -1.38)	-1.16(-1.26, -1.06)	-1.88(-2.00, -1.76)
**Mortality rate**	-0.59(-0.65, -0.51)	-0.67(-0.72, -0.59)	-0.57(-0.62, 0.51)	-2.10 (-2.22, -1.98)	-1.94(-2.08, -1.81)	-2.30(-2.52, -2.08)
**DALYs rate**	-0.77(-0.84, -0.71）	-0.83(-0.93, -0.72)	-0.72(-0.77, -0.66)	-2.04(-2.16, -1.92)	-1.87(-2.00, -1.74)	-2.25(-2.36, -2.14)
**YLLs rate**	-0.80(-0.87, -0.74)	-0.84(-0.96, -0.76)	-0.76(-0.81, -0.70)	-2.12(-2.24, -1.99)	-1.96(-2.09, -1.82)	-2.32(-2.54, -2.09)
**YLDs rate**	0.42(0.32, 0.52)	0.43(0.31, 0.54)	0.32(0.26, 0.38)	-1.48(-1.58, -1.38)	-1.13(-1.25, -1.02)	-1.89(-2.01, -1.76)

Supplementary Table 4 Incidence, Deaths, and DALYs of various cancers in the world regions in 2019, with 95% uncertainty intervals (UI) in Female

Cancer type	Incidence			Deaths			DALYs		
	Number	Rate per 10,000	Change in rates(%)	Number	Rate per 10,000	Change in rates(%)	Number	Rate per 10,000	Change in rates(%)
Acute lymphoid leukemia	11259 (8777-14034)	1.77 (1.38-2.2)	-0.09 (-0.59-0.71)	4373 (3276-5772)	0.69 (0.51-0.91)	-0.43 (-0.73-0.11)	374421 (279981-494908)	27.9 (20.87-36.88)	-0.42 (-0.72-0.14)
Acute myeloid leukemia	4008 (3108-5396)	0.63 (0.49-0.85)	-0.17 (-0.56-0.5)	2028 (1606-2664)	0.32 (0.25-0.42)	-0.29 (-0.65-0.32)	173641 (137005-228469)	12.94 (10.21-17.03)	-0.28 (-0.64-0.34)
Brain and central nervous system cancer	13215 (9935-15742)	2.07 (1.56-2.47)	-0.11 (-0.61-0.36)	6066 (4643-7162)	0.95 (0.73-1.12)	-0.34 (-0.7-0.03)	517633 (396029-611664)	38.58 (29.51-45.58)	-0.33 (-0.69-0.05)
Hodgkin lymphoma	724 (560-855)	0.11 (0.09-0.13)	-0.01 (-0.3-0.71)	190 (126-242)	0.03 (0.02-0.04)	-0.43 (-0.61-0.01)	16112 (10795-20532)	1.2 (0.8-1.53)	-0.42 (-0.6-0.04)
Kidney cancer	5480 (4680-6333)	0.86 (0.73-0.99)	-0.04 (-0.32-0.26)	954 (814-1101)	0.15 (0.13-0.17)	-0.31 (-0.5--0.11)	83874 (71561-96871)	6.25 (5.33-7.22)	-0.29 (-0.49--0.08)
Lip and oral cavity cancer	273 (223-332)	0.04 (0.03-0.05)	0.22 (-0.06-0.61)	73 (58-91)	0.01 (0.01-0.01)	0.06 (-0.22-0.44)	6065 (4874-7521)	0.45 (0.36-0.56)	0.08 (-0.2-0.47)
Liver cancer	1100 (801-1459)	0.17 (0.13-0.23)	-0.43 (-0.59--0.24)	1151 (867-1459)	0.18 (0.14-0.23)	-0.49 (-0.66--0.28)	99106 (74391-125290)	7.39 (5.54-9.34)	-0.48 (-0.65--0.27)
Malignant skin melanoma	856 (413-1716)	0.13 (0.06-0.27)	0.15 (-0.11-0.82)	102 (65-181)	0.02 (0.01-0.03)	-0.21 (-0.41-0.26)	9114 (5682-16222)	0.68 (0.42-1.21)	-0.19 (-0.39-0.3)

Nasopharynx cancer	190 (164-219)	0.03 (0.03-0.03)	-0.24 (-0.41--0.05)	59 (48-72)	0.01 (0.01-0.01)	-0.54 (-0.66--0.38)	4914 (4048-5958)	0.37 (0.3-0.44)	-0.53 (-0.65--0.36)
Non-Hodgkin lymphoma	2704 (2090-3541)	0.42 (0.33-0.56)	-0.02 (-0.25-0.3)	1377 (1166-1614)	0.22 (0.18-0.25)	-0.44 (-0.6--0.12)	116858 (98713-136527)	8.71 (7.36-10.17)	-0.43 (-0.59--0.11)
Other leukemia	25965 (18971-33211)	4.07 (2.98-5.21)	-0.69 (-0.81--0.39)	3765 (2609-4948)	0.59 (0.41-0.78)	-0.77 (-0.85--0.55)	338224 (232952-443773)	25.21 (17.36-33.07)	-0.77 (-0.85--0.54)
Other malignant neoplasms	22020 (19034-25601)	3.46 (2.99-4.02)	-0.05 (-0.24-0.2)	8529 (6997-10522)	1.34 (1.1-1.65)	-0.31 (-0.45--0.09)	735329 (604814-906584)	54.8 (45.07-67.56)	-0.29 (-0.44--0.08)
Ovarian cancer	989 (788-1227)	0.16 (0.12-0.19)	0.56 (-0.18-1.27)	201 (157-257)	0.03 (0.02-0.04)	0.31 (-0.35-1.01)	16896 (13282-21588)	1.26 (0.99-1.61)	0.34 (-0.34-1.06)
Thyroid cancer	711 (587-875)	0.11 (0.09-0.14)	0.21 (-0.07-0.67)	39 (32-47)	0.01 (0.01-0.01)	-0.29 (-0.45-0.11)	3578 (2924-4364)	0.27 (0.22-0.33)	-0.24 (-0.41-0.17)

Note: Data in parentheses are 95% Uncertainty Intervals (95% UIs); DALYs Disability-Adjusted Life Years；SDI = sociodemographic index.

Supplementary Table 5 Incidence, Deaths, and DALYs of various cancers in the world regions in 2019, with 95% uncertainty intervals (UI) in Male

Cancer type	Incidence			Deaths			DALYs		
	Number	Rate per 10,000	Change in rates(%)	Number	Rate per 10,000	Change in rates(%)	Number	Rate per 10,000	Change in rates(%)
Acute lymphoid leukemia	15353 (11543-18520)	2.26 (1.7-2.72)	-0.04 (-0.42-0.77)	7000 (4972-8633)	1.03 (0.73-1.27)	-0.34 (-0.6-0.22)	598389 (424625-737620)	41.75 (29.62-51.46)	-0.32 (-0.59-0.26)
Acute myeloid leukemia	5568 (4025-7288)	0.82 (0.59-1.07)	-0.12 (-0.41-0.74)	3244 (2358-4279)	0.48 (0.35-0.63)	-0.18 (-0.45-0.71)	277961 (201255-367396)	19.39 (14.04-25.63)	-0.16 (-0.45-0.76)
Brain and central nervous system cancer	17104 (11945-21151)	2.51 (1.76-3.11)	-0.2 (-0.57-0.44)	8972 (6253-11118)	1.32 (0.92-1.63)	-0.35 (-0.65-0.18)	766818 (535679-950168)	53.5 (37.37-66.29)	-0.34 (-0.64-0.21)
Hodgkin lymphoma	1689 (1173-2117)	0.25 (0.17-0.31)	-0.24 (-0.45-0.26)	675 (436-899)	0.1 (0.06-0.13)	-0.44 (-0.6--0.03)	56232 (36369-74701)	3.92 (2.54-5.21)	-0.43 (-0.59--0.01)
Kidney cancer	5513 (4590-6567)	0.81 (0.67-0.97)	0 (-0.27-0.44)	1470 (1131-1854)	0.22 (0.17-0.27)	-0.2 (-0.42-0.19)	128123 (98491-161602)	8.94 (6.87-11.27)	-0.18 (-0.41-0.21)
Lip and oral cavity cancer	187 (154-227)	0.03 (0.02-0.03)	-0.05 (-0.32-0.33)	61 (49-75)	0.01 (0.01-0.01)	-0.17 (-0.42-0.2)	5078 (4087-6217)	0.35 (0.29-0.43)	-0.16 (-0.41-0.22)
Liver cancer	1103 (812-1494)	0.16 (0.12-0.22)	-0.26 (-0.46--0.03)	1208 (923-1506)	0.18 (0.14-0.22)	-0.36 (-0.56--0.13)	103894 (79297-129924)	7.25 (5.53-9.06)	-0.35 (-0.56--0.11)
Malignant skin melanoma	569 (326-1830)	0.08 (0.05-0.27)	0.42 (-0.14-1.06)	110 (68-232)	0.02 (0.01-0.03)	-0.19 (-0.46-0.27)	9626 (5871-20217)	0.67 (0.41-1.41)	-0.16 (-0.45-0.3)

Nasopharynx cancer	227 (192-271)	0.03 (0.03-0.04)	-0.31 (-0.5--0.02)	86 (71-103)	0.01 (0.01-0.02)	-0.56 (-0.68--0.35)	7113 (5902-8585)	0.5 (0.41-0.6)	-0.54 (-0.67--0.33)
Non-Hodgkin lymphoma	5177 (4090-6657)	0.76 (0.6-0.98)	-0.09 (-0.3-0.25)	2613 (2198-3094)	0.38 (0.32-0.45)	-0.39 (-0.55--0.04)	221540 (187086-261916)	15.46 (13.05-18.27)	-0.38 (-0.54--0.02)
Other leukemia	23340 (18711-30338)	3.43 (2.75-4.46)	-0.63 (-0.78--0.24)	4031 (3171-5474)	0.59 (0.47-0.8)	-0.73 (-0.84--0.45)	357917 (283056-486364)	24.97 (19.75-33.93)	-0.72 (-0.83--0.43)
Other malignant neoplasms	19448 (16094-23208)	2.86 (2.37-3.41)	-0.12 (-0.37-0.22)	11560 (9206-14365)	1.7 (1.35-2.11)	-0.35 (-0.51--0.1)	992716 (789636-1231181)	69.26 (55.09-85.9)	-0.34 (-0.5--0.09)
Thyroid cancer	436 (362-519)	0.06 (0.05-0.08)	0.46 (0.16-0.81)	48 (39-58)	0.01 (0.01-0.01)	-0.14 (-0.34-0.09)	4158 (3385-4957)	0.29 (0.24-0.35)	-0.1 (-0.32-0.14)
Acute lymphoid leukemia	15353 (11543-18520)	2.26 (1.7-2.72)	-0.04 (-0.42-0.77)	7000 (4972-8633)	1.03 (0.73-1.27)	-0.34 (-0.6-0.22)	598389 (424625-737620)	41.75 (29.62-51.46)	-0.32 (-0.59-0.26)

Note: data in parentheses are 95% Uncertainty Intervals (95% UIs); DALYs Disability-Adjusted Life Years；SDI = sociodemographic index.
